# A Viable and sustainable flat- membrane plate-and-frame module for spent acid regeneration and metal ion recovery

**DOI:** 10.1016/j.heliyon.2023.e18344

**Published:** 2023-07-17

**Authors:** Shazia Perveen, Syed Ghazanfar Hussain, Muzamil Jalil Ahmed, Ruba Khawar, Taha Bin Siraj, Maryam Saleem

**Affiliations:** a Department of Chemistry, NED University of Engineering & Technology, University Road, Karachi 75270, Sindh, Pakistan

**Keywords:** Diffusion dialysis, Ion exchange membranes, Metal ion recovery, Spent acid regeneration, Quaternized polyepichlorohydrin

## Abstract

This study provides techno-economical insights for acid regeneration and metal recovery from spent acidic wastewater by a diffusion dialysis plate-and-frame module using Quaternized Polyepichlorohydrin – Polyacrylonitrile (QPECH-PAN) membranes. Quaternized Polyepichlorohydrin (QPECH) membranes were synthesized using 1,4-diazobicyclo[2.2.2]octane (DABCO) and blended with polyacrylonitrile (PAN). Said membranes were analyzed in terms of their mechanical, physicochemical, and electrochemical characteristics, providing significant results comparable to the commercial membranes (IEC: 1.76 mmol/g, SD: 60.91%, Permselectivity: 79.5 ± 0.31%, and transport no. t_(−)_: 0.5). Mechanical characterization reveals that the QPECH-PAN membranes possess comparable mechanical strengths (tensile strength: 329.56 MPa). Further, sheet resistivity (6.11 Ω cm^2^) and conductivity (0.16 S/cm^2^) reveal the relative conductive nature of these membranes. Percent acid recovery and metal ion recovery ratios were found to be 72% and 48% respectively, and separation factors were 126.8 and 84.57 respectively. The QPECH-PAN membrane's techno-economic feasibility was also analyzed within the context of a textile industry processing up to 5500 kg/d of acidic wastewater. It indicates a potential cost saving of US $0.53 million on H_2_SO_4_ and NaOH, as well as an OPEX saving of 40.91% against a semi-continuous acid neutralizer.

## Highlights

• Low-cost self-supporting membranes based on Quaternized Polyepichlorohydrin (QPECH) and Polyacrylonitrile (PAN).
• A single stack flat membrane plate-and-frame module indicating H_2_SO_4_ recovery upto 72% and Fe^2+^ ion recovery by 48%.
• High conductivity and adequate ion exchange performance for diffusion dialysis and potential electrodialysis applications.
• Techno-economic evaluation of the QPECH AEM indicates an attractive short investment recovery of 11.4 months.
• An OPEX saving of 40.91% against a semi-continuous acid neutralizer.

## Graphical abstract

**Figure:**
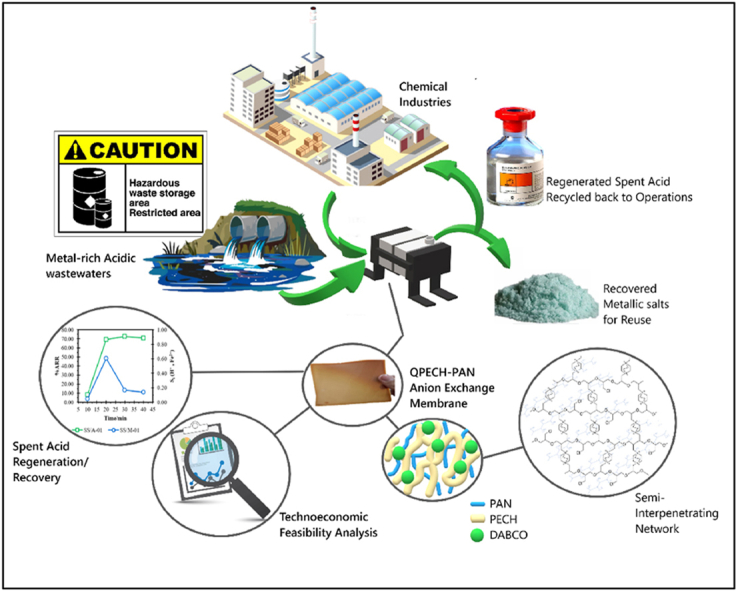

## 1 Introduction

The textile, petroleum, petrochemical, hydrometallurgical, semiconductor and many other industries produce highly acidic wastewater from etching, pickling and bleaching processes. These are rich in a variety of inorganic acids, salts, caustics, heavy metals and sulfidic content. The textile industry is among the largest polluters and generates up to 350 kg of acidic wastewater per 100 kg of fabric processed [[1], [2], [3]]. Meeting environmental regulations and standard requisites are key ethical, social and governance (ESG) considerations for industries. This has led to investments in wastewater treatment plants and waste recovery units [4]. However, the complications in procuring wastewater treatment plants owing to rising economic uncertainty and downturn are proving too discouraging. Often, industries have allegedly discarded untreated acidic run-offs and metal-rich tailings [5]. Regulations mandating sustainability practices and environmental protection have led to a significant shift towards low-waste production [6]. Many industries have been considering acidic and metal-rich wastewater treatment in the developing world, although much progress still needs to be made, owing to cost constraints and technical infeasibilities.

Conventional methods used for acidic wastewater treatment include solvent extraction [[7], [8], [9], [10]], ion exchange processes [[11], [12], [13], [14]], reverse osmosis [15,16], catalysis [17,18], activated carbon processes [19,20], chemical and electrochemical processes [[21], [22], [23], [24], [25]] and by biomass/biosorption [26]. Though these methods are effective for waste water treatment but only a few are barely used by the industrial sector for economic and technological reasons [27,28]. For example, most of the textile industry plants treat their acidic waste water with Ca(OH)_2_, NaOH or KOH [6,29,30]. Notably, these acidic wastewater treatments generate by-product/precipitate solid wastes that add to environmental hazards and operating cost burdens of cash-strapped industries. To this end, chemical recycling such as spent acid regeneration (sulfuric/chloric) seems a lucrative option to save costs on such capital- and resource-intensive processes.

Industrial applications have been exhibit increasing use of ion-exchange membranes for the last three decades, and have proven to be reliable and economically beneficial [31]. Its applications typically involve separation processes, electricity generation, fuel cell development, flow batteries, water purification and desalination processes [[32], [33], [34]]. As compared to other traditional methods, membrane technology is simple, cost-effective, environment friendly and employed without the production of by-products. Dead-end membrane separation technologies such as plate-and-frame diffusion dialysis (DD) and electrodialysis (ED) [34] have been adopted for the recovery of caustics, acids, sulphides, and metal ions from waste waters. DD has been assumed to be a most optimal process for spent acid regeneration/recovery for high concentration feeds >0.5 M [35].

Kim et al. reported the use of Asahi Polysulfone (APS) membrane in a modified DD for the recovery of H_3_PO_4_ and Al^3+^ from etching wastewater [36]. AEMs such as Selemion DSV, AMX, Fuji A, Neosepta AFN, FumaSep® (Q-SPEEK) have been extensively used by industries for spent acid regeneration and metal ion recovery [[37], [38], [39]]. The researchers above have primarily focused on the process optimization of (spent) acid recovery/regeneration using commercial and modified commercial membranes. Despite the attractive performance characteristics of such membranes, these have been known to require frequent corrective maintenance which contributes to higher operational costs for the chemical industry [35].

AEMs must provide an adequate trade-off between cost and performance. The use of speciality polymers (such as quaternized polysulfones, polyethyl ether ketone and polyphenylene oxide) and their chemical modifications, coating, composite preparations are typical strategies to enhance anion exchanging performance [[40], [41], [42]]. However, this has frequently led to complicated and capital-intensive materials that are not sustainable for the long-term. Moreover, these must withstand long operational cycles and severe operating conditions [35]. In this regard, Polyepichlorohydrin (PECH) derivatives could be considered which could resolves said issues. Although, considered for power generation, desalination and fuel cell applications, these membranes exhibit remarkably versatile physio-chemical and electrochemical characteristics [37,41,[43], [44], [45]]. Further these membranes can exhibit anti-fouling properties [37]. The attractiveness of the material is further increased by the appreciable production cost and greenness involved in its preparation [46].

In this work, the incremental research herein attempts to explore the properties and performance capabilities of QPECH-PAN membranes for potential versatile applications including spent acid regeneration and metal ion recovery. The prepared membranes are self-supporting and would thus eliminate costs associated with the use of expensive support materials like PTFE, PEEK, and other materials as with previous reported composite membranes [43,47]. We further propose a new resistivity/conductivity measurement technique based on four probe co-linear DC resistivity analysis, which was not reported previously for these membranes.

It is of note that current literature regarding the use of this membrane in the field of spent acid regeneration and metal ion recovery is scarce. However, Gong et al. (2023) and Chen et al. (2023) have recently studied different types of QPECH membrane's performance in acid recovery via DD [48,49]. The QPECH membrane used, however, does not utilise a cyclic diamine unlike our work. More so, the use of QPECH-PAN AEM has not been explicitly considered for DD-based acidic textile wastewater treatment. For this purpose, we designed a single-stack flat membrane plate-and-frame module, utilizing a flat sheet QPECH-PAN AEM for the spent H_2_SO_4_ regeneration and Fe^2+^ recovery. Moreover, the techno-economic feasibility of this membrane will also be studied in this paper, against acidic wastewater treatment processes in current use by textile industries.

## 2 Materials & methods

### 2.1 Materials

Sodium chloride (NaCl), Potassium Permanganate (KMnO_4_; >99.8% pure) were purchased from Merck kGaA (Darmstadt, Germany). UHPLC-MS grade deionized water was used (Carlos-Erba, La Vadreuil, France) to minimize the chances of impurities, during synthesis and analytical processes of QPECH-PAN membranes. Polyepichlorohydrin (PECH) (M_w_ = 700,000 by GPC),1,4-diazobicyclo[2.2.2]octane (DABCO) (98% purity), Dimethyl sulfoxide (DMSO) (98% purity), *N*,*N*-dimethyl formamide (DMF) (98% purity) and Polyacrylonitrile (PAN) were purchased from Sigma Aldrich. Paraffin Oil BP-grade was purchased from Karachi Pharma, Pakistan. Demineralized water (commercial-grade; pH 7.1) was used for the plate-and-frame module experiments.

### 2.2 Synthesis & fabrication of QPECH AEMs

**Fig. 1:**
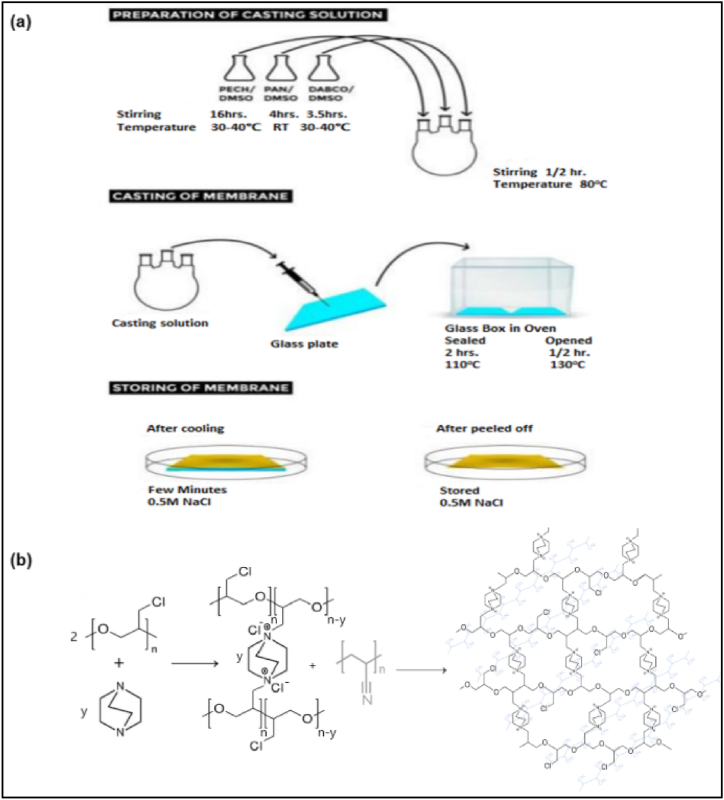
Schematic of Synthesis of Anion Exchange Membrane and Quaternization Reaction of Polyepichlorohydrin with 1,4-diazobicyclo[2.2.2]octane, followed by the creation of the sIPN matrix by Polyacrylonitrile addition.

Synthesis of the quaternized polyepichlorohydrin (QPECH) Anion Exchange Membranes (AEM) was done based on Güler et al. (2012) work [44]. The synthesis procedure involves a quaternization reaction in between a cyclic diamine, 1,4-diazobicyclo[2.2.2]octane (DABCO) and chloromethyl-bearing active polymer, Polyepichlorohydrin (PECH) (Fig. 1a and b). Details regarding the procedure for QPECH synthesis and membrane preparations are provided in Supplementary Information, S.I., Appendix A, S1–S2. The mass-based PECH to PAN blend ratio ‘σ’ and the excess amine ratio ‘*v*’ were calculated from Eqs. (Eq. 1), (Eq. 2), as under:

(Eq. 1) $$\sigma =\frac{m_{PECH}}{m_{PAN}}\text{;}$$

(Eq. 2) $$v=\frac{n_{diamine}}{n_{{CH}_{2}Cl}}\text{;}$$

The thickness δ (in microns, μm) of the synthesized QPECH-PAN membrane was measured using the conventional micrometer screw gauge (Digital Micrometer, 0–25 mm range, ±0.003 mm error). The thickness of the dry membranes was measured to be 100–133 μm. Other QPECH-PAN membranes (see Table 1) were also prepared with different blend ratios and amine ratios using the above method and their ion-exchanging properties have been discussed in detail, in results Section 3.1 of this paper. QPECH-PAN membranes have been labelled as “QP/A-n” and “QP/B-n” where A and B are the blend and amine ratios respectively and ‘n’ is the membrane number.

**Table 1:** Physicochemical parameters with varying blend ratios (“QP/A series”).

*Membranes*	σ (g/g)	*v* (g/g)	δ (μm)	*S.D. (%)*	IEC (mmol/g)^a^	FCD (mmol/g H_2_O)	ε	λ
QP/A-01	0.1	0.5	67	20.09	1.33	6.62	0.28	8.39
QP/A-02	0.2	0.5	91	33.81	1.7	5.03	0.40	11.05
QP/A-03	0.3	0.5	77	43.42	1.71	3.94	0.46	14.11
QP/A-04	0.4	0.5	115	49.27	1.76	3.57	0.49	15.55
QP/A-05	0.5	0.5	126	53.63	1.75	3.26	0.51	17.03
QP/A-06	0.6	0.5	133	60.91	1.76	2.89	0.54	19.23
QP/A-07	0.7	0.5	154	65.04	1.80	2.77	0.56	20.07
QP/A-08	0.8	0.5	178	72.71	1.83	2.52	0.59	22.07
QP/A-09	0.9	0.5	164	76.61	1.86	2.43	0.60	22.88

a Dry membrane base-weight, for all PAN/QPECH AEMs equal to 1.945 g/cm^2^ average.

### 2.3 Membrane characterization and physicochemical properties

#### 2.3.1 Physicochemical characterization

Details regarding the physicochemical characterization of the membrane are provided in Supplementary Information, S.I., Appendix A, S3. These characterizations include the argentometric ion exchange capacity (IEC) determination and the evaluation of dry-wet properties (swelling degree, bound water molecules, and membrane void porosity) of the QPECH-PAN membranes. Replicate measurements were taken to minimize experimental transience.

#### 2.3.2 Fourier-transform infrared spectroscopy

The synthesized QPECH-PAN membranes were analyzed to evaluate their chemical functionalities using a Shimadzu FTIR-ATR Prestige IP-21 (Kyoto, Japan) spectrometer, equipped with an attenuated total reflectance (ATR) flat-tip attachment. Membrane samples were dried and cleaned and subsequently pressed onto the diamond crystal. No further sample preparation was needed.

#### 2.3.3 Permselectivity (α) and transport number (t_+/−_)

The synthesized QPECH AEM membranes' permselectivity ‘α’ was analyzed by the electromotive force (emf) or Hittorf's method described elsewhere [44]. The two-compartment cell was filled with 0.5 and 0.1 M KCl instead. Further, two Ag/AgCl (1 M/KCl) reference electrodes were inserted into the solutions close to the membrane connected to a potentiostat (Biologic SP200 with EC-Lab v11.10 software). The potential drop was monitored with the aid of a couple of Ag/AgCl reference electrodes. After 1 h of stirring of the solution with magnetic stirrers, the potential drop between two electrodes was measured as ‘E_mem_’ and the permselectivity (α) was calculated using Equation (3).

(Eq. 3) $$\alpha =100\frac{E_{mem}}{E_{th}}\text{;}$$

Where, ‘E_th_’ is the membrane potential for a 100% permselective membrane, determined using the Nernst Equation (4).

(Eq. 4) Eth=Eo−RTnFln⁡(a1a2);

Where ‘R’ is the universal gas constant (8.314 J mol^−1^ K^−1^), ‘F’ is the Faraday constant (96,500 C mol^−1^), and ‘T’ is the temperature (298 K), ‘n’ is the number of moles of charges involved, ‘a_1_’ and ‘a_2_’ are compartment solution activities for the KCl solutions at the given concentrations. Hittorf's method was further used to determine the transport number of a membrane [44]. The transport number is estimated from the membrane potential (or voltage drop) ‘E_mem_’ determined above. The transport number for the co-ion (i.e., Cl^−^), ‘t*_(−)_’, was estimated using Equation (5).

(Eq. 5) t(−)*=12+EmemF2RTln⁡(C1C2);

Where, ‘t*_(−)_’ is the transport number of the counter ion, ‘R’ is the universal gas constant, ‘F’ is the Faraday constant, and ‘T’ is the temperature. ‘C_1_’ and ‘C_2_’ are compartment solution concentrations in molars.

#### 2.3.4 Surface characterization

##### 2.3.4.1 Field emission scanning electron microscopy (FE-SEM)

FE-SEM samples were prepared from dried and cleaned membranes. Membrane was cut into a square of 1 × 1 mm film. Sample was subsequently sputtered with a thin layer of gold using a JEOL JFC-1500 (Tokyo, Japan) sputterer for 10–15 min under a 0.1 atm vacuum. The synthesized membrane (QPECH) was examined by using the JEOL JSM6380A (Tokyo, Japan) SEM instrument. Images were taken at 5 kV, across varying magnification ranges (250× - 12,000×) at the surfaces of the membrane.

##### 2.3.4.2 Atomic force microscopy (AFM)

For AFM analysis 1 × 1 mm^2^ of synthesized QPECH-AEM film was cut and the Agilent 5500 AFM system (USA) was used for AFM imaging using the ‘tapping’ mode with the maximum scanning range of 90 μm × 90 μm × 8 μm. The AFM system was equipped with Si_3_N_4_ triangular probe and images were obtained and presented by the built-in Pico View 1.12.2 Software. Surface roughness parameters as defined by ISO 25178 were determined using the same software.

#### 2.3.5 Pore size analysis

The pore size analysis was further performed using the conventional permeate velocity test for the plate-and-frame module via the Guerout-Elford-Ferry Equation (6). The detailed procedure has been reported elsewhere [50,51]. The pore profiles assumed under this equation are finger-like nanopores that have been observed to exist for typical heterogeneous ion exchange membranes [51,52].

(Eq. 6) $$r_{m}=\sqrt{\frac{8\delta Q\eta \left(2.9-1.75\varepsilon \right)}{\varepsilon A\Delta P}}\text{;}$$

Where, ‘r_m_’ is the membrane pore radius (in metres), ‘δ’ is the membrane thickness, ‘Q’ is the permeate flow-rate, ‘η’ is the water viscosity at 27 °C (0.00086 Pa s), ‘ε’ is the membrane void porosity determined above, ‘A’ the effective membrane area (49 cm^2^) and ‘ΔP’ the net operating (feed) pressure (0.1 MPa). Porosity measurements and pore size distributions were evaluated using the semi-automated image processing technique proposed by Rabbani et al. (2017) [53] using the MATLAB® R2021a software. The pore size estimation was compared with the method described above and was adjusted as per the specifications of the FESEM equipment used in this study.

### 2.4 Membrane resistivity and conductivity

The dried and cleaned synthesized QPECH-PAN membrane (1 cm × 3.5 cm, 0.13 μm thick) was analyzed using a four-point co-linear resistivity measurement technique as described elsewhere [54]. The resistivity measurement was performed at an applied current 1 mA DC using a four-point probe system, namely the Everbeing EB-6V mmW Optimized Probe station, Taiwan) equipped with a data acquisition module Keithley SCS-4200 (USA) with KITE software (see Supplementary Information. S.I., Appendix B, S1). These adjustable probes are 5 μm dia. Tungsten needles. These probes were placed onto the membrane film in a co-linear fashion, equidistant to each other (by 1 mm each). This instrument can measure the surface resistance among different pairs of needles adjusted at the thin membrane. The resistance ‘R’ for a membrane of area ‘A’ having length ‘L’ and width ‘W’, is given by Equation (7).

(Eq. 7) $$R=\rho \frac{L}{A}=\overset{\rho_{s}}{\overset{︷}{\left(\frac{\rho }{t}\right)}}\frac{L}{W}\text{;}$$

Where, ‘ρ_s_’ is the sheet volume resistivity (Ω. sq) is also given as:

(Eq. 8) $$\rho_{s}=\frac{\pi }{\ln\left(2\right)}\left(\frac{V}{I}\right)\text{;}$$

**Fig. 2:**
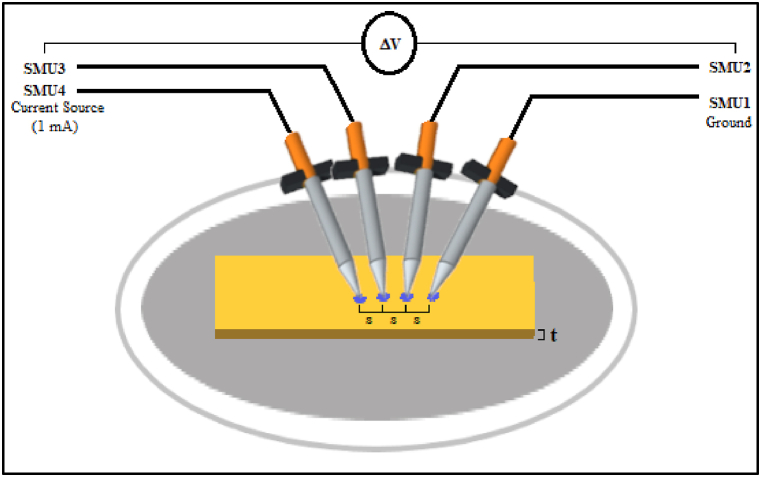
Model configuration for the four probes co-linear bulk resistivity and conductivity analysis. Where,‘s’ is the probe spacing (1 mm, equidistant), ‘t’ is the membrane thickness; SMU refers to the source-measurement unit and GNDU is the ground unit.

Where ‘V’ is the measured voltage (V) and ‘I’ is the source current (A). Eq. (8) is valid when the sample film/membrane thickness is less than half the probe spacing, i.e., t < ^s^/_2_ [55] where ‘s’ is probe spacing, the distance between probes. Eq. (7) can be inverted to get conductivity ‘σ_s_’ (S/sq) and Eq. (8) to get conductance (S/sq). Sheet resistance and conductivity can be multiplied and divided by membrane thickness ‘t’ to get resistance and conductivity respectively. The configurational setup is shown in Supplementary Information. S.I., Appendix B S1. Data of measured bulk resistance ‘R_avg_’ and conductivity ‘S_avg_’ is given is also provided therein. The probe array is centered around the material, as shown in Fig. 2.

### 2.5 Mechanical strength and integrity

The tensility (tensile strength) ‘σ_B_’ of the synthesized (QPECH) membrane was determined using ZwickRoell Z005 TN Proline Material Testing Machine (Germany). The tensile test samples (2 cm × 8 cm, 133 μm thickness) were cut from a dried and wet membrane (soaked in 0.5 M NaCl for >72 h). After cleaning with cotton wipes, the sample was gripped between the clamps at a nominal gauge length of 10 mm and gauge width of 15 mm. The displacement rate was 5 mm/min, conducted at room temperature (27.5 °C). A testXpert II software was used to plot the static stress-strain curve. Young's modulus ‘E’ can also be determined from the slope (rise vs. run) just before the elongation break ‘ε_B_’. The high SD% of the QP/B membrane series (see Table 2) meant that their structural integrity and mechanical strength was not sufficient to conduct tensility tests (at *v* > 0.5). Further, the QP/A membranes (having 0.1 ≤ σ < 0.3) had poor mechanical strengths characterised by high brittleness.

**Table 2:** Physicochemical parameters with varying amine rations (“QP/B series”).

*Membranes*	σ (g/g)	*v* (g/g)	δ (μm)	*S.D. (%)*	IEC (mmol/g)^a^	FCD (mmol/g H_2_O)	ε	λ
QP/A-06^b^	0.6	0.5	133	60.91	1.76	2.89	0.54	19.23
QP/B-01	0.6	2.6	158	78.0	1.79	2.29	0.60	24.21
QP/B-02	0.6	3.4	182	77.6	1.78	2.29	0.60	24.22
QP/B-03	0.6	4.2	126	80.42	1.83	2.28	0.61	24.41
QP/B-04	0.6	5.2	136	83.63	2.33	2.79	0.62	19.94

a Dry membrane base-weight, for all PAN/QPECH AEMs equal to 1.945 g/cm.^2^ average.

b QP/A-06 serves as the starting point for variation in amine ratios.

### 2.6 Diffusion dialytic recovery using simulated spent metal-rich acidic run-offs

**Fig. 3:**
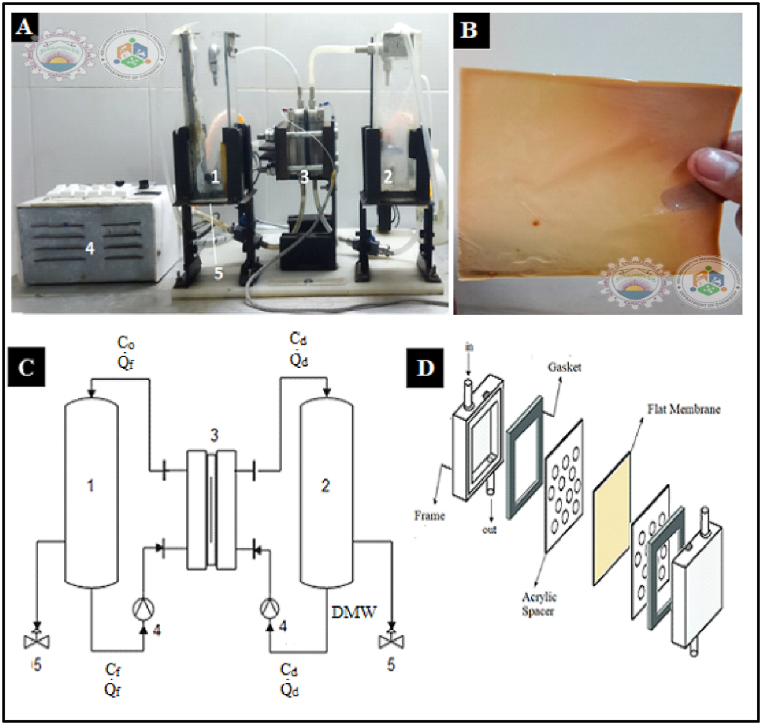
(A) Single flat membrane plate-and-frame module (B) Synthesized QPECH AEM (C) PFD (Process Flow Diagram) of the module (D) Membrane sandwich assembly. Note: DMW is demineralized water; C_f_, C_d_, and C_o_ are the feed, diffusate and outlet concentrations in mol/L; Q_f_, and Q_d_ are feed and diffusate flow-rates in L/hr.

On a bench-top basis, acidic and metal recovery from simulated acidic and metal-rich waste solution was done using the diffusion dialysis (DD) process. Further, on a medium lab-scale setup, the DD process was performed. The single 10 × 10 cm flat membrane plate-and-frame module was designed (Fig. 3A) using the prepared QPECH-PAN membranes (Fig. 3B). The single-stack module comprises a single sheet of QPECH-PAN membrane separating into one feed (retentate) cell and another diffusate cell. The effective area of the membrane for diffusional dialysis is about 64 cm^2^, whereas the total membrane area is about 100 cm^2^ (10 cm × 10 cm).

The membrane sandwich assembly (Fig. 3D) comprises the QPECH-PAN membrane which is sandwiched between polymethyl methacrylate spacers and acid-resistant rubber gum gaskets. One of the two compartments was filled with simulated feed solution, “SS/x-n” (where, “SS” is the simulated wastewater solution, “x’” is acid “A” and/or metal “M”, “n” is the sample number corresponding to different concentrations) and the other compartment was filled with demineralized water. Mechanical stirring was performed to achieve concentration polarization. The experimental time for the single cycle was 30 min and titrimetric analysis was performed periodically. The pH and conductivities of solutions were measured using Jenway 4330 bench-top pH/conductivity meter (Cole-Palmer; UK).

Both the feed column ‘**1**’ and the receiving column ‘**2**’ were of 1.2 L capacity, and were constructed from plexiglass. The membrane sandwich ‘**3**’ (Fig. 3C) was sealed with a rubber sealant via hydraulic press. Controllable centrifugal pumps ‘**4**’ were used and a feed flow-rate ‘Q_f_’ of 4.2 L/h and a diffusate flow-rate ‘Q_d_’ of 2.4 L/h were maintained. Samples were drawn from the valves ‘**5**’ after every 15 min from the two columns. For a run-time of 1 h, an optimum recovery for at least 400 mL feed was obtained. The recovery of the desired component (acid, and/or metal) was determined via titrimetric analysis (see Supplementary Information, S.I., Appendix A, S4).

Different tests were conducted for analysis of acid (i.e., H^+^) and metal (i.e., Fe^2+^) recovery from simulated waste solutions. Percent component recovery ratio $\left(\eta_{i}\right)$ and dialysis coefficient $\left(U_{d,i}\right)$ were determined by acid and Fe^2+^ concentrations at outlet/return waste stream Co,i (i = H^+^, Fe^2+^) and those at the outlet stripping stream, Cd,i (i = H^+^, Fe^2+^). Percent component recovery ratio was calculated using Equation (9) [39,56].

(9) $$\eta_{i}=\frac{C_{d,t}Q_{d}}{C_{f,t}Q_{f}}\text{;}$$

Where, ‘Cf,t’ and ‘Cd,t’ correspond to the nominal concentration of acid or Fe^2+^ in the feed and diffusate at operation time ‘t’ respectively, Qf and Qd are feed and diffusate flow-rates in L/hr. The apparent dialysis coefficients Ud,i in L/m^2^. hr can be calculated using Equation (10) [39,56].

(10) $$U_{d,i}=\frac{M_{i}}{A\Delta C_{i}}\text{;}$$

Where, Mi is the molar flow-rate (mol/hr) of the component transported across the effective area ‘A’ of membrane in m^2^. ‘ΔCi’ is the log mean concentration difference of the component across the membrane in mol/L. Equation (11) was used to calculate ΔCi.

(11) $$\Delta C=\frac{C_{f}-C_{d}-C_{o}}{\ln\left(\left(C_{f}-C_{d}\right)/C_{o}\right)}\text{;}$$

The outlet (returned) acid or Fe^2+^ concentration ‘Co’ (in mol/L) and outlet stripping acid or Fe^2+^ concentration ‘Cd’ (in mol/L), were determined using the following Equations (12), (13) [39,56].

(12) $$C_{o}=\left(1-\eta \right)C_{f}\text{;}$$

(13) $$C_{d}=\frac{Q_{f}}{Q_{d}}\eta C_{f}\text{;}$$

Where, ‘Si’ is the separation factor or the measure of selectivity of the prepared QPECH AEM towards Fe^2+^ or H^+^, ‘Ud,d’ and ‘Ud,r’ are the diffusate and retentate dialytic coefficients. Si can be estimated using Equation (14) [56].

(14) $$S_{i}=\frac{U_{d,r}}{U_{d,d}}\text{;}$$

## 3 Results & discussion

### 3.1 QPECH-PAN AEM synthesis, characterization and performance evaluation

AEMs are typically synthesized in a three-step process, where the active polymer backbone is first chloromethylated using chloroalkyl alkyl ethers, then subsequently quaternized using long-chain sec- or tert-amines. Adopting the strategy of Güler and co-workers, a one-pot “two-reaction, one reagent” strategy can be followed which can drastically reduce time-, labor- and energy input into the synthesis of the AEMs [44]. The use of the versatile, relatively cheaper, relatively safer diamine quaternization and cross-linking reagent such as DABCO is much more cost-effective and greener. The preparation and blending of Quaternized Polyepichlorohydrin (QPECH) elastomers with immobilized linear polymers such Polyacrylonitrile (PAN), gives way to highly cross-linked and intercalated polymer matrices, where the latter penetrates deep into the former's matrix [57]. This creates extensive cross-linked networks, called semi-interpenetrating networks (sIPNs) known for their high chemical compatibility, thermo-mechanical strength, and electrochemical characteristics [58,59]. These properties can have significant implications for high-performance features for diffusion dialysis membranes.

Fig. 1 Shows quaternization/cross-linking reaction in between tertiary amine, DABCO, and an active polymer, PECH. An inert polymer, such as Polyacrylonitrile (PAN) was also introduced as a “conditioner” or strength-enhancer into the membrane to increase tensility and mechanical strength. Generally, PECH-DABCO membranes are fragile, brittle, and prone to tearing [60]. Therefore, it is required to use any crosslinking agent [61] or conditioning polymers [62]. Additionally, porous supports made of polytetrafluoroethylene (PTFE), polyethyl ether ketone (PEEK) [47] or meshes/fabric of inert materials can also be used to add further mechanical strength. The prepared QP/A-x membranes are self-supporting as evidenced by their mechanical strength in the following section.

#### 3.1.1 Physicochemical characterization

Membrane performance in any electrochemical and industrial operation depends on the type of the membrane [44,63] and the physicochemical and electrochemical properties of the membranes [32,37,64,65]. IEMs possess different properties such as ion exchange capacity (IEC), swelling degree (SD), fixed charge density (FCD), bound water per ionic group (λ), membrane void porosity (ε), permselectivity (α), transport number (t*) and many other electrochemical properties which may be affected by the change in its constitutional composition. Table 1, Table 2 Show the results of QPECH-PAN membranes performance at varying blend ratios and amine ratios.

**Fig. 4:**
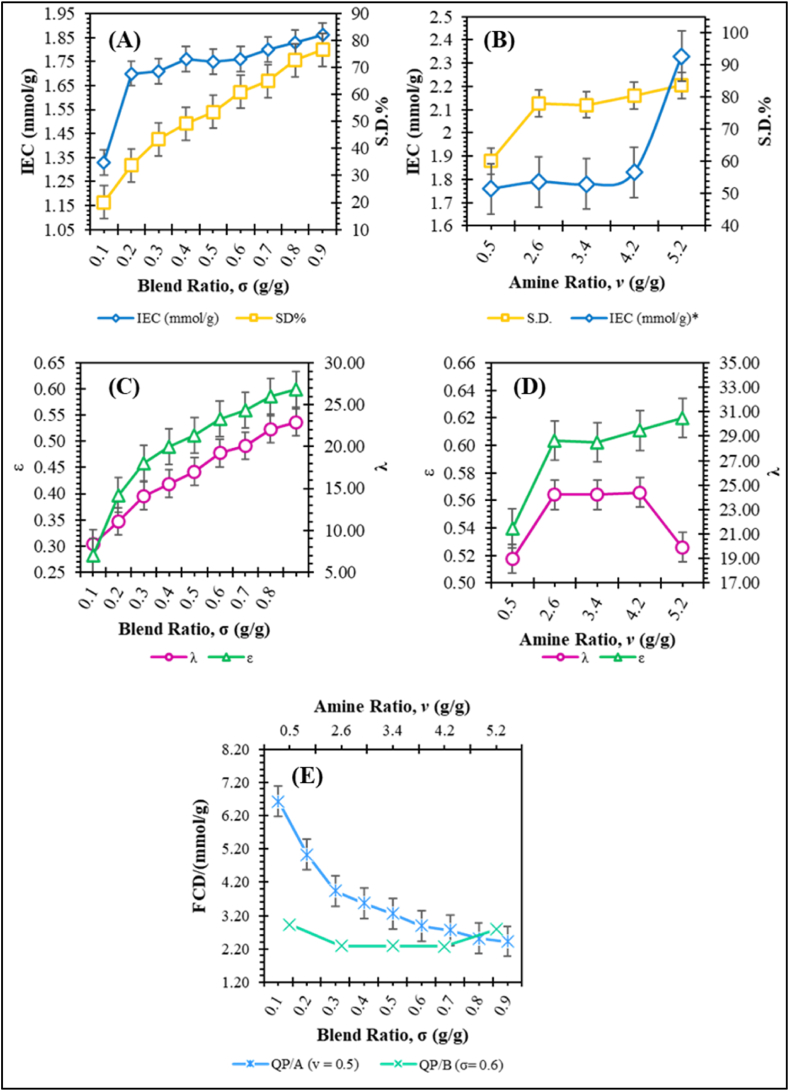
Trends followed by the parameters relating to ion exchange performance for various PAN/QPECH AEMs.

As evident from Table 1., an increase of the blend-ratio ‘σ’ leads to an increase in the IEC and SD% of the PAN/QPECH AEMs (see Fig. 4A). Further, the hydrophobicity of such membranes tends to decrease due to the influence of PAN over PECH [66,67]. PAN is said to be relatively more hydrophilic owing to the polar –CN group [68]. In other words, this leads to increase in ion exchanging and water uptake property of the PAN/QPECH AEM, as reported elsewhere [44].

Increasing IEC and SD% may also be attributed to the influence of the blended PAN with the quaternary amine (DABCO) groups in the PECH backbone, at that composition, owing to the competing behaviour between the labile PECH backbone and DABCO diamine within the sIPN membrane matrix. This also indicates that the water of hydration or bound water per ionic group (λ) also increase correspondingly, which is a crucial property needed for AEMs to resist fouling [68]. Thus, an optimum blend and amino ratio must be selected to ensure the best mechanical and ion exchanging performance.

With increasing amine ratio ‘*v*’, a stable trend is generally followed across all membranes until a sharp increase in IEC at 5.2 g/g. SD% shows relatively constant trends with increasing amine ratios (see Fig. 4B and Table 2). Further, the membrane void porosity ‘ε’ and the bound water molecules per ionomer group ‘λ’ are inversely proportional to each other for both QP/A and QP/B series.

The FCD is relatively stable around 2.2–3.2 for varying amine ratios Fig. 4E., whereas, the varying blend ratios indicate a gradual decline in FCD. Further for the QP/B membrane series, at *v* > 4.7, the FCD begins to increase. QP/A membranes likely exhibit such trends because of the competing behaviour between PECH and DABCO during the quaternization reaction [41]. Moreover, the membrane begins to swell less with increasing formation of the sIPN network (internal crosslinking) in QPECH-PAN membrane matrix [58]. The ‘λ’ is the highest for QP/A-03, followed by QP/A-06, where the –CH_2_Cl groups of PECH are quaternized with equivalent diamines of DABCO, i.e., when σ = *v* = 0.5–0.6. These parameters follow the trends of IEC and SD% respectively (see Fig. 4C–D).

Considering the results above, it is evident that **QP/A-06** possesses optimum ion exchange capacity, desired physicochemical properties and minimal PAN-DABCO interference. **QP/A-06** was selected as the best membrane at which optimum performance can be achieved. The results of this membrane are in good agreement with the literature [44]. **QP/A-06** membrane was considered for further characterization and diffusion dialysis experiments.

#### 3.1.2 Fourier transform infrared (FTIR) spectroscopy

**Fig. 5:**
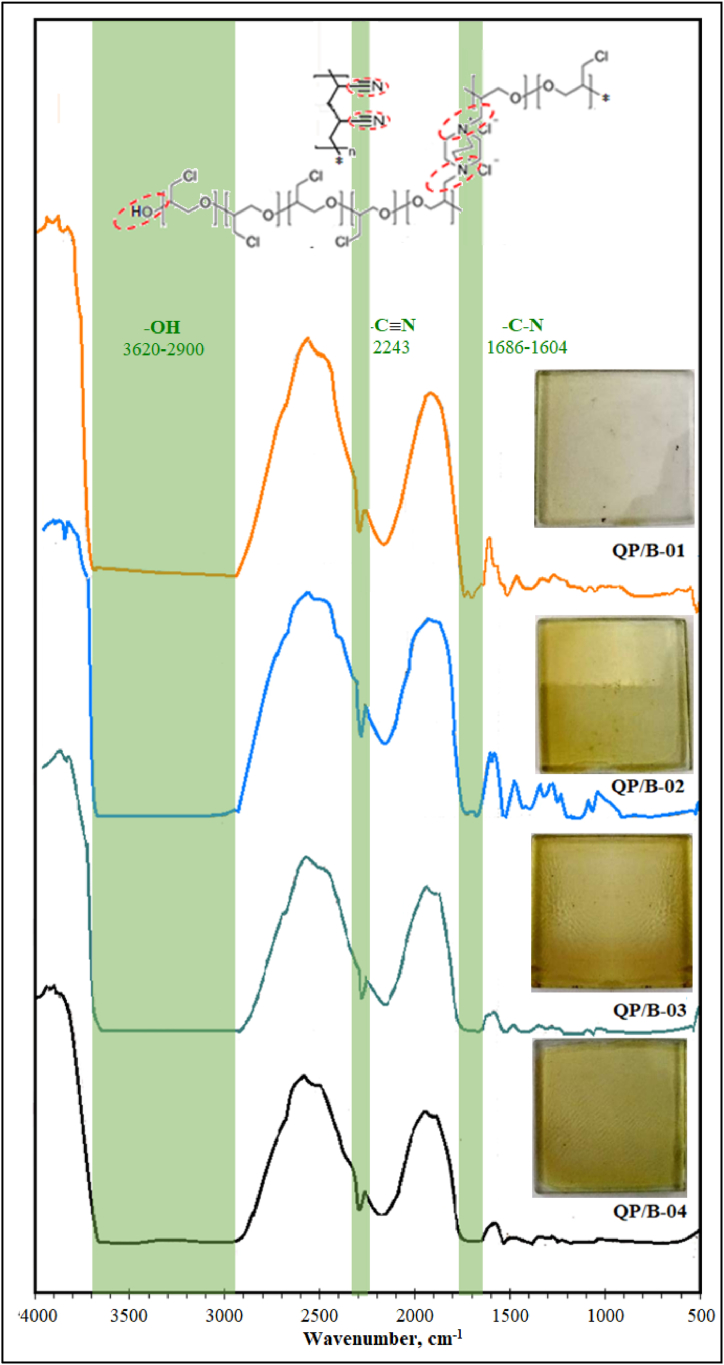
FTIR Results of the synthesized PAN/QPECH AEM with varying amine ratios: QP/B-01 (2.6), QP/B-02 (3.4), QP/B-03 (4.2), and QP/B-04 (5.2). Inset: The chemical structure of PAN/QPECH AEM as well as the physical forms of the casted membranes.

To find the optimum amine ratio, FTIR spectra of QP/B-01 to QP/B-04 were recorded (Fig. 5) which indicate the increase in % transmittance of quaternized group (-C-N: 1686–1604 cm^−1^) with the increase in amine ratio. Amination process is responsible to develop ion exchange sites at active polymer. Thus, increasing the amine ratio, ion exchange groups in the membrane per PECH monomer, was observed to increase. It also leads broadening of the –OH peak, which implies that the hydrophilicity (and thus, the water uptake) of these membranes also tends to increase. The * symbol in the PAN moiety implies the continuance of the PAN monomers, which may create a cross-linked web-like structure (not shown), surrounding the QPECH chain. FTIR data associated with PAN/QPECH AEMs prepared with varying blend ratios can be found elsewhere [44].

**Fig. 6:**
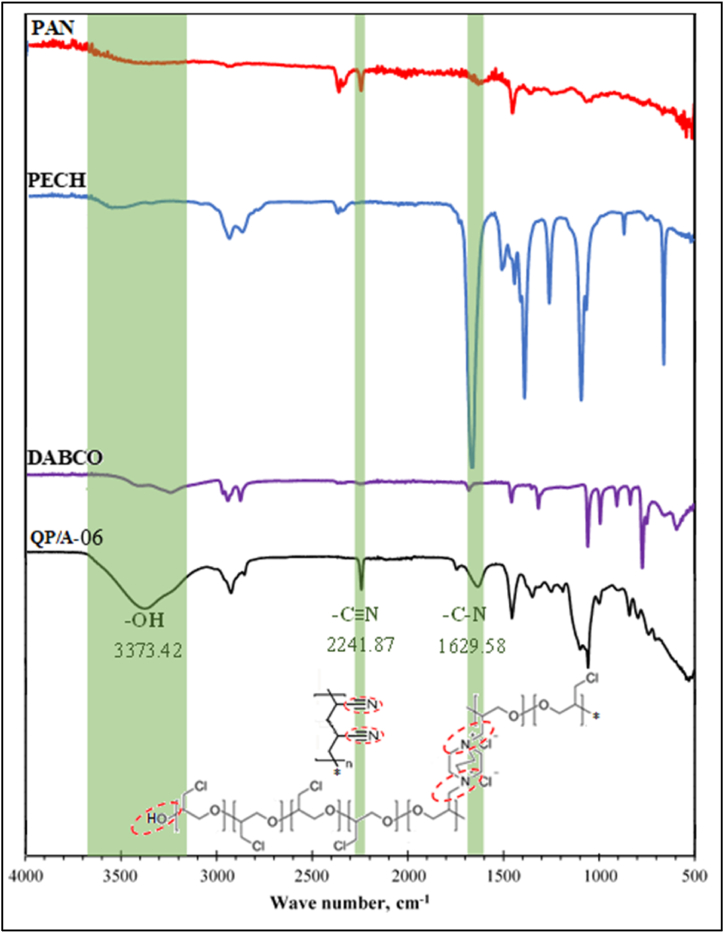
FTIR Results of the selected, synthesized PAN/QPECH AEM (“QP/A-06”) and its constituents. Inset: The chemical structure of PAN/QPECH AEM with the distinguishing peaks (and functional groups) highlighted and encircled.

Further, FTIR spectra of the QPECH-PAN membrane and its constituents is given in Fig. 6. A sharp peak at 2241.87 cm^−1^ due to the nitrile group of the PAN conditioner can be observed, which confirms the PAN blending. Furthermore, the high degree of the blend ratio (0.6) also contributes to the sharp response. A broad peak at 3373.42 cm^−1^ can also be observed which can be attributed to the terminal –OH group of the PECH active polymer. The quaternization (C–N linkage) is also confirmed by the 1629.58 cm^−1^ stretching peak and 1100–1073 cm^−1^ bending peaks. Results obtained agree with that reported by Güler et al. (2012) [44].

#### 3.1.3 Permselectivity (α) and transport number (t*_(−)_)

Permselectivity indicates the selective transport of counter-ions of IEMs and the ability to exclude co-ions. The permselectivity, α, for the prepared QPECH AEM (QP/A-06), was determined to be 79.5 ± 0.31%, at an observed membrane potential ‘E_mem_; of −3.41 V. The obtained permselectivity is in good compliance with the values reported by Güler et al. (2012) [44]. Permselectivity is related to the transport of electric charges. The transport number quantifies the fraction of the charge carried by the specific (counter) ions. The transport number for the Cl^−^ ion, t*_(−)_, was determined to be 0.489 (or t_(+)_: 0.511), which was found in good agreement with the reported data (see Table 5). A high permselectivity (>70%) is generally required for high power output and high recovery applications in ED and DD designs respectively [37,44]. To construct a waste recycling stack of high performance, QPECH-PAN membranes must possess low resistance, high permselectivity, good mechanical, and firm stability with low degree of swelling or shrinking. The properties of membranes required for high performance often counteract with each other.

#### 3.1.4 Surface characterization

**Fig. 7:**
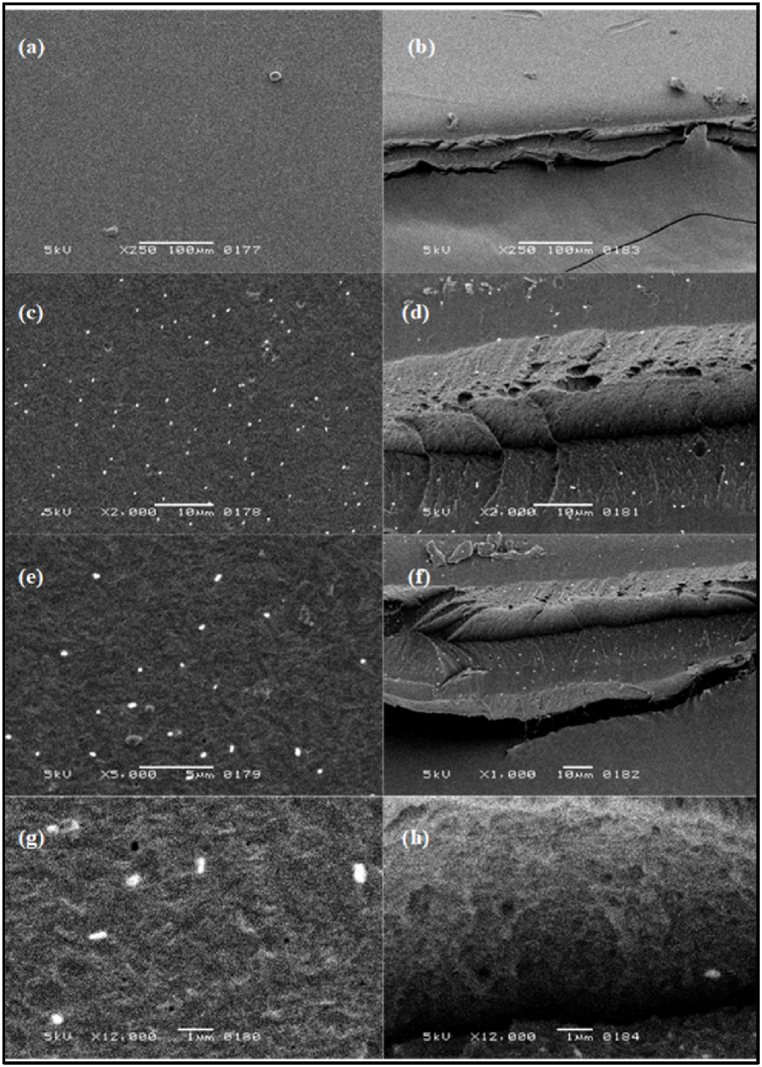
FE-SEM Images of the synthesized PAN/QPECH AEM taken at 5 kV at (a–b) 250×, (c–d) 2,000×, (e) 5,000× (f) 1,000× and (g–h) 12,000×.

The FE-SEM images of the synthesized QPECH AEM (QP/A-06) are shown in Fig. 7(a-h) across varying magnifications (250× to 12,000×) revealing a relatively smooth surface of this membrane. The implications of having an adequate smoothness to the surface of the membrane imply that for the present QPECH-PAN AEM (QP/A-06), the negatively charged surface would lead to higher repulsive force against potential foulants with negative charges. Thus, a smoother surface indicates a lower susceptibility towards membrane fouling owing to lower contact areas. For regions, characterised by low shear forces, the fouling layers will also have a lower likelihood to form [69].

**Fig. 8:**
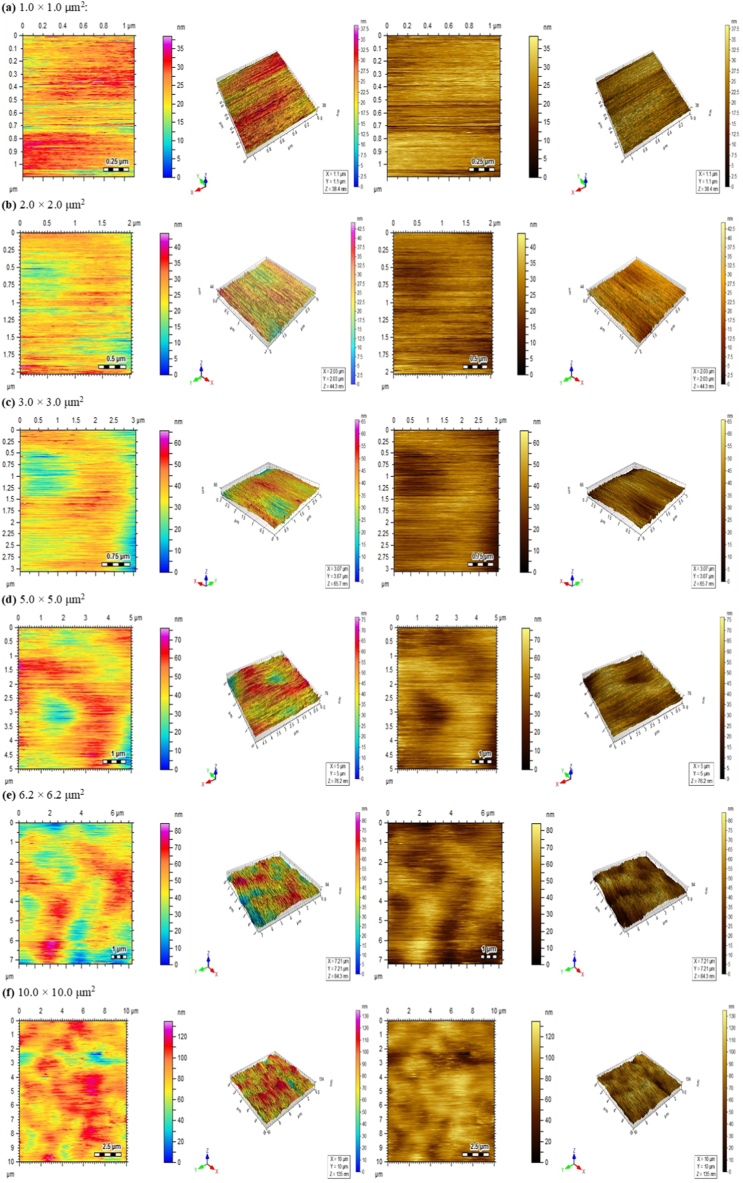
AFM images (phase and topographical image sets are shown in 2D and 3D) of the QPECH AEM (QP/A-06).

Fig. 8 present the atomic force microscopy (AFM) phase and topographical images of the synthesized QPECH AEM surface. The membrane surface was smooth with smaller size peaks and valleys. This assumption is supported by the determined values of the ISO 25178 roughness parameters, i.e., the root-mean-square roughness ‘Sq’, the skewness ‘Ssk’ and the kurtosis ‘Sku’, as tabulated in Supplementary Information. S.I., Appendix B, S5. The −1<Ssk<1 (but Ssk ≠ 0) indicates that the surface height distributions of the QPECH AEM membrane are skewed from the mean, and the Sku values are approximately to 3.0 corresponding to a normal distribution. Although such a Sku value implies that sharp portions and indented portions co-exist, it can be observed that these values are in the nano-scale which means the synthesized membrane is smooth.

Ion exchange membranes possess smooth surfaces with roughness in the nanoscale [44,62]. Further, it can be observed in the AFM images (Fig. 8a–f), that pores were not discernible using this technique. Membranes that are used in diffusion dialysis (DD), electrodialysis (ED), or similar applications are not considered to be typically non-porous, as they possess a labile polymer matrix that in relation to the SD%, can alter the pore size and shape [70]. Nanopores have been known to exist in ion exchange membranes, with pore sizes ranging from as much as 6 nm–10^4^ nm [71]. These nanopores are typically formed by the modifiers such as PAN and solvents such as DMSO, used in the membrane preparation of the QP/A-06.

#### 3.1.5 Pore size analysis

The pore formation and deformation in the present membrane is due to the interactions between the polar and non-polar components of membrane matrix, namely PECH and DABCO. This induces shear-stresses that can result in pore formations/deformations. Similar mechanisms are also reported for other ion exchange membranes [71]. The influence of aqueous electrolytes or water can lead to swollen states that pull apart polymeric chains. Hydration of water clusters with the ionomer groups (QPECH) of the membrane further contribute to the formation of micro, nano or mesopores [52]. Interfacial stresses between PECH and DABCO can lead to swollen states in the presence of water, during the phase inversion of the QPECH-AEMs.

The permeate velocity tests provided an average pore size, r_m_ of 77 nm of the QP/A-06 membrane (δ = 133 μm) at a pure water flux of 0.086 L/m^2^. hr at an applied pressure of 0.1 MPa. The average pore size, r_m_, reflects the radius of the finger-like nanopores [51]. Furthermore, based on the work of Rabbani et al. (2017) [53], the surface images were processed using the SEM Image Porosity & Pore Size semi-automated image processing algorithm (See Supplementary Information. S.I., Appendix B, S2). The pore size and porosity estimations are tabulated in Table 3, as under.

**Table 3:** Porosity calculations obtained using the SEM Image Porosity & Pore Size semi-automated image processing algorithm (surface images only).

Fig #	Magnification	Average Pore Radius (μm)	Porosity^*1*^	Standard Deviations
a	250×	0.0062	0.0703	0.0013
c	2,000×	0.0078	0.1830	0.0025
e	5,000×	0.0085	0.2105	0.0032
g	12,000×	0.0088	0.2702	0.0034

**Note:**^*1*^A difference between porosity and membrane void porosity ‘ε’ (free solution volume within a wet membrane, indicated in Table 1, Table 2), was observed as FESEM sputtering covers the voids in the membranes thereby reducing void size (pore size and dimension). A spatial resolution of 3.0 nm/pixel was fixed for the JEOL JSM6380A SEM.

Results from the Rabbani's SEM Image Porosity & Pore Size semi-automated Image Processing Algorithm indicate that the average membrane pore size is 75 ± 13 nm for the analyzed QP/A-06 membrane surface. The pore size is comparable to that which has been determined using the Guerout-Elford-Ferry equation (eq. (11)) from the results of the water permeability testing and swelling degree. The porosity (0.17 ± 0.1) estimated from this technique is three times less than that determined using the permeate velocity test (dry-wet method [72]). In FESEM sample preparation, gold sputtering (or any metal coating) can lead to the ‘masking’ or ‘burying’ of smaller nanopores, reducing their effective void volumes [51]. The reduction in pore sizes can be attributed to the decrease in membrane roughness and the aggregate sizes atop its surface.

### 3.2 Mechanical strength and integrity of QPECH AEMs

**Fig. 9:**
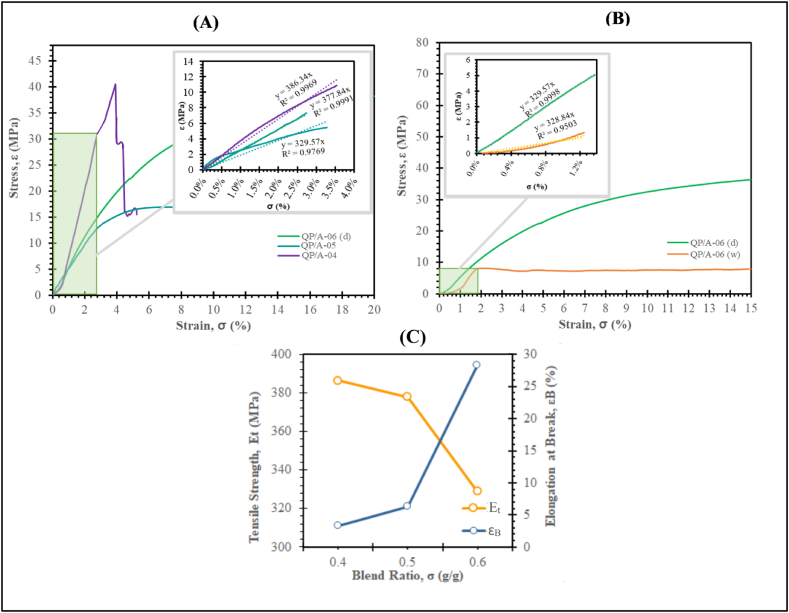
(a). Stress-strain curve of varying QPECH-PAN AEM blends (“QP/A series”), and **(b)** Stress-Strain Curves for the wet and dried QP/A-06 AEMs. Insets: Linear part of the stress-strain curves (Note, the slope of the regression equals the Young Modulus).

AEMs used in diffusion dialysis (DD), electrodialysis (ED), or similar applications must possess excellent mechanical strength to withstand pressure drops due to high flow-rates. Tensile strength and elongation at break are two important parameters that indicate the general mechanical performance of AEMs [73]. The results of stress-strain measurement for the QP/A membranes are given in Fig. 9a. QP/A-04 indicated high brittleness with higher E_t_ value (377.84 MPa), indicated that the PECH-DABCO crosslinking (sIPN) formation is sub-optimal and are dominated by the higher concentration of the PAN modifier added. QP/A-06 and QP/A-05 indicate ductility higher E_t_ values (329.54 MPa and 386.34 MPa, respectively), inferring adequate and optimized sIPN network formation. The increase in PAN concentration in the QP/A membranes (low ‘σ’) leads to higher E_t_ and ε_B_ for the blend membranes [74]. This trend is also evident in Fig. 9a, where lowering PAN concentration leads to lower E_t_ and ε_B_ for the QP/A membranes.

Results indicate the increased swelling reduces mechanical strengths of the QP/A membranes, owing to the sIPN network. Similar effects have also been observed for other membranes that exhibited cross-linking and tightly “knit” assemblies [75,76]. The increase in PECH (higher σ) leads to higher degree of crosslinking with DABCO and the formation of the QPECH sIPN networks. Ultimately, the ‘tightness’ and extensive crosslinking between PECH-DABCO impedes the mobility of supramolecular ionomer chains within the membrane, leading to higher E_t_ and lower ε_B_ values at lower blend ratios (see Fig. 9c).

The sIPN features of the QPECH-PAN AEMs have been observed to provide a relatively good and comparable mechanical performance with most commercial membranes under dry conditions, as shown in Table 6. From Fig. 9b, It is obvious that the blend ratio (0.6) used, has contributed to the excellent tensility (or Young's Modulus) ‘E_t,d_’ (329.54 MPa) for the ‘dry’ QP/A-06 membrane. The stress at break ‘σ_B_’ (25.5 MPa) and elongation at break ‘ε_B_’ (28.3%) of the dry membrane, were relatively high despite a low thickness (133 μm). Whereas, for the same membrane of similar thickness yet soaked in 0.5 M NaCl for >72 h, the tensility was reduced by ca. 0.2% (E_t,w_ = 328.84 MPa). The wet QP/A-06 also indicated a lower ‘σ_B_’ (6.5 MPa) and an increased ‘ε_B_’ (42.7%). The insignificant loss in mechanical strength after storage in wet or either dry conditions is not adverse, and shows that the QP/A-06 is mechanically capable for DD processes during operations.

### 3.3 Areal resistivity and conductivity of QPECH AEM

Conventional membrane resistance analysis is based on AC techniques such as EIS [40,77,78] or LCR [79]. AC resistance is more than its DC resistance owing to the *skin effect*, i.e., the non-uniform distribution of an AC flowing through a conductor and its tendency to concentrate near the surface of the conductor at a certain level or skin depth (the current density is ca. 37% at the surface). The skin depth depends on the current frequency and the conductor's (here the QP/A-06) electromagnetic properties [80]. The result is an increase in effective resistance.

Surface conductivity and areal resistance are significant characteristics of IEMs that can be employed in electrochemical applications [37,44]. In the present work, we report a simple, facile DC technique for the sheet resistance and conductivity analysis of the thin film membrane. The authors of this work would like to add that no DC method of analysis based on our proposed technique has thus far been reported in AEM literature. Areal resistance ‘r’ and conductivity ‘σ’ were found to be 6.11 Ω cm^2^ and 0.157 S/cm^2^ respectively. The Sheet resistivities and conductivities are provided in Supplementary Information. S.I., Appendix A, S6.

Compared to conventional membranes, like Brexar 70, Nafion 115, Nafion 117, HDX 100 and FumaSep, the conductivity of the QP/A-06 is higher by 63.7%, 12.7%, 85.9%, 89.5, 94.9% respectively. Although Nafion membranes are of the cationic-type, their use has also been claimed and industrially adopted in spent acid regeneration and metal recovery [81,82]. The high conductivity of the QP/A-06 can be attributed to the high charge distributions enabled by the sIPN characteristics of the membrane. Similar characteristics are also reported in the literature [45].

Surface conductivity (or resistance) is a significant parameter for AEMs that corresponds to the surface charge transfer rate across a flat sheet or thin-film membrane. Conductivity (or resistance) can be used for studies related to anti-bacterial, electrocatalytic, and fouling behavior and in sensor applications. Bulk resistivity can be used to quantify the electromagnetic field generated by applying a constant DC to moderately or highly conductive AEMs [44]. This electromagnetic field generated by AEMs is known to decrease fouling, owing to the electrostatic repulsion of charged particles in the feed or by increased diffusive mobility of charged particles atop the surface. Surface resistance is dependent on the membrane thickness as well as the membrane resistance [83]. These properties are significantly useful in electrochemical fuel cells, electrodialysis and reverse electrodialysis units.

Area resistance is a characteristic property of membranes, referring to the resistance per unit area of the membrane that may be impacting ion transport/exchange [37]. Higher area resistance infers that the membrane would be promoting lower ion transport/exchange. Membranes having high permselectivity may inevitably lead to higher area resistance which is not desirable for DD and ED applications [44]. The relation between membrane thickness and area resistance is proportional, as thick membranes have high area resistance. Therefore, it is often challenging to maintain the minimum thickness for low resistance and to compromise mechanical stability. Thus, an optimum blend and amine ratio must be selected to ensure the best mechanical and ion-exchanging performance.

Moreover, for asymmetric membranes, surface conductivities infer that how the surface layer facilitates ionic mobility. Surface structure, swelling and pore characteristics, lead to higher electric conductivity at membrane surface [84]. Ionic conductivities can be higher for both at the surface and in the bulk of membrane. Notably, a key difference lies in the manner and the extent to which ion mobility is promoted by membrane structure [85].

### 3.4 Diffusion dialytic recovery using simulated spent metal-rich acidic run-offs

**Fig. 10:**
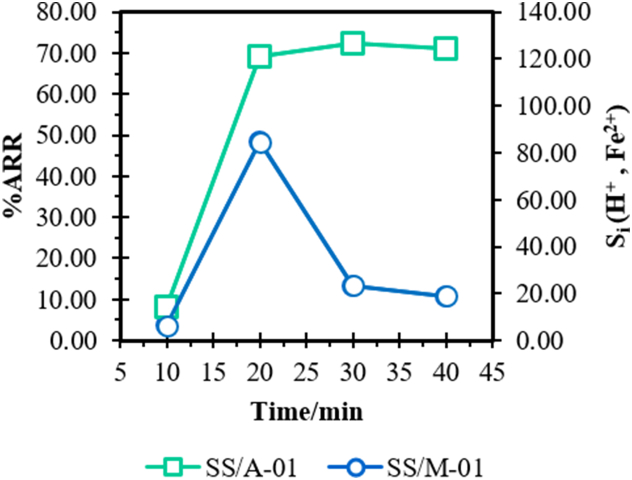
Acid/Fe^2+^ recovery ratio and separation factor of the DD operation using the prepared QPECH AEM, of SS/A-01 (1.05 M H_2_SO_4_) and SS/M − 01 (0.03 M FeSO_4_.7H_2_O/H_2_SO_4_) by the single flat membrane plate-and-frame module.

The diffusion dialysis experiment data is available in the Supplementary Information. S.I., Appendix A, S4. The estimated DD parameters have been tabulated in Table 4. All experiments were conducted in RTP conditions (25 °C). Fig. 10 shows the pictorial view of diffusion dialytic performance of prepared QPECH AEM.

**Table 4:** Performance data of the DD Acid/Metal recovery by the single flat membrane plate-and-frame module using the prepared PAN/QPECH AEM.

	t (min)	ΔC (g/L)	U_d,r_ (L.m^2^/hr)	U_d,d_ (L.m^2^/hr)	S_i_	η_i_ (%)	VEF
***SS/A-01****1.05 M H*_*2*_*SO*_*4(aq)*_	10	66.130	58.35	8.34	14.29	8.16	1.61
	20	35.799	48.76	59.00	121.00	69.14	0.54
	30	32.925	33.38	42.33	126.80	72.46	0.48
	40	33.043	23.11	28.71	124.21	70.98	0.51
							
***SS/M-01****0.03 M FeSO*_*4*_*.7H*_*2*_*O/H*_*2*_*SO*_*4*_	10	7.37	56.76	3.53	6.22	3.56	1.69
	20	4.09	37.80	31.96	84.57	48.33	0.90
	30	8.49	18.58	4.32	23.27	13.30	1.52
	40	6.27	12.98	2.47	19.00	10.86	1.56

Note: SS/x-n, where “SS” is the simulated wastewater containing acid “A” or metal “M”. Whereas, “n” is the sample no. corresponding to different concentration. ‘ΔC’, ‘U_d,d_’, ‘U_d,r_’, ‘S_i_’ and ‘η_i_’ are the log concentration difference, the diffusate and retentate dialytic coefficients, the separation factor, and the percent component recovery ratio, respectively, at operation times ‘t’.

Acid and Fe^+^ recovery as well as the separation factor against the DD time was measured. It was noted that for the simulated acid drainage sample, SS/A-01 and metal-rich sample SS/M − 01, maximum ‘η_i_’ and/or ‘S_i_’ is achieved at 30 and 20 min of operation, respectively. Maximum recovery of 72% (with S: 126.8) was observed for SS/A-01, whereas recovery of 48% (with S: 84.57). Further operation results in insignificant recovery as the acid and Fe^2+^ content become constant with time. Recovery has been optimized at a feed and diffusate flow-rate of 4.2 and 2.4 L/h for the specified effective membrane area.

The demineralized water (DM) to acid/Fe^2+^-rich feed charge flow ratio, Q_d_/Q_f_, was optimized at 0.6, as the maximum acid/Fe^2+^ recovery ratio was achieved using this flow ratio. While a high incline in flow ratio implies an increased contact time and increased diffusion rate. This can, however, lead to recovered component dilution. Changes in membrane hydrophilicity and surface structure contribute to the relative decrease in recovery during DD operation [39,56,86,87]. Based on the three-phase membrane model, as discussed thoroughly in previous studies [87,88], counter-ions “hop” through the active region whereas the co-ions “leak” through the interstitial zone of IEMs, owing to the low repulsion.

**Fig. 11:**
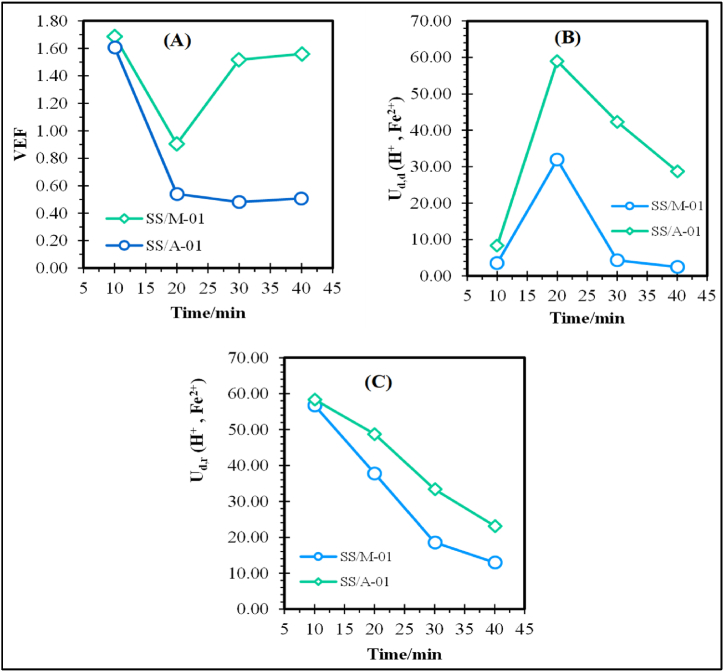
A Estimated volumetric expansion factors, and diffusate dialysis coefficients (L/m^2^. hr), for **11B.** SS/A-01 (1.05 M H_2_SO_4_), and **11C.** SS/M − 01 (0.03 M FeSO_4_.7H_2_O/H_2_SO_4_).

For relatively high swelling degree (or water uptake) of the membrane, the hydrated ions will begin to migrate through the membrane, permit a higher permeability of the counter-ion (here, SO_4_^2−^) and significantly increase the transport of co-ions (here, H^+^ and Fe^2+^). It is of worth mentioning that the co-ion (H^+^ and Fe^2+^) transport is limited by the membrane water uptake as it lowers gradually at higher DD times. Thus, the separation factor (or selectivity) of H^+^ to Fe^2+^ ions tends to become constant after increasing gradually. Higher permselectivity (here, 79.5 ± 0.31%) for AEM would also lead to faster counter-ion transport. The volumetric expansion factor (VEF) is another significant membrane property that affects DD operational performance. A high VEF not only increases the pumping energy (“burden of recycling” [89]) but leads to a decrease in selectivity. A detailed study of VEF across the DD operation time for QPECH AEM has been done and results are provided in Fig. 11(a–c), and in Table 4. VEF was determined using Eq. (15).

(Eq. 15) $$VEF=\frac{C_{o}}{C_{d}}\text{;}$$

The VEF-DD time relation tends to decrease to near unity at 20 min for system SS/A-01 (1.05 M H_2_SO_4_) and seemingly returns to initial VEF values beyond this time. For the Fe^2+^-rich acidic simulated wastewater (“SS/M-01”), the VEF-DD time increased after 30 min of DD operation. Lowering the recycling burden has been reported at VEF >1.0 [39]. VEF-DD time relations may be changed due to the competition in between hydrated co-ions and water molecule transport to waste (return) stream, using the three-phase membrane model [87,88]. The DD operation up to 20 min is ill-advised for system SS/A-01, as it leads to higher pumping energy demand. This may be attributed to the limiting water uptake levels with increasing DD time and can be attributed to the cross-linking effect (the sIPN nature) of the membrane which at optimum conditions hinders the water transport through the outlet waste (return) stream.

More so, the apparent diffusate (acid/Fe^2+^) dialysis coefficient ‘U_d,d_’ also tends to decrease (Fig. 11B) with DD time. The slow decrease in concentration of feed leads to increase in acid/Fe^2+^ dialysis coefficients. Considering the optimum PECH/PAN blend ratio ‘σ’ (0.6) and amine ratio ‘*v*’ (0.5), the achieved SD% (60.1%) for the QPECH AEM used will enhance the permeability and transport rate of counter-ion (here, SO_4_^2−^) and co-ions (here, H^+^ and Fe^2+^). These membrane properties coupled with the specified optimum DD operating time (ca. 20–30 min) may contribute to the probable lowering burden of recycling and maintain the desired separation efficiency (or selectivity).

### 3.5 Comparison of QPECH AEM performance

A comparative analysis of QPECH anion exchange membrane with commercially available membranes has been done and results are given in Table 6, Table 5, Table 7. Our synthesized membrane, QPECH AEM (QP/A-06), was found to possess much better results of IEC and SD as compare to commercially available membranes. However, the permselectivity of QP/A-06 membrane is comparable to some of the commercially available membranes. It is well known that ion exchange membranes show counteracting properties [37], for example membranes with a high swelling degree possess a low fixed charge density, low permselectivity and low area resistance. It means membranes possessing high permselectivity might show high area resistance, which is not desired at commercial scale. Therefore, optimization of the membrane characteristics for commercial applications is quite a challenging task. However, our membrane exhibits excellent results of other separation indicators such as the dialysate/diffusate coefficient and the separation factors, in comparison to its peers.

**Table 5:** Parameters of AEMs related to Ion Exchange Performance. All membranes were tested at 25 °C (RTP), unless specified otherwise in parentheses.

	δ(μm)	*α* (%)	*SD. (%)*	IEC (mmol/g)	*t*^a^_(−)_	U_d,d_ (L/m^2^.hr)	S	Ref.
*QPECH-PAN (QP/A-06)*	133^a^	79.5 ± 0.31	60.91	1.76	0.489	31.96–42.86 (37.80 for Fe^2+^)	121.0–126.8	Present Work
***PECH-C***	77.0	79.2 ± 0.90	53.5 ± 0.20	1.88 ± 0.01	–	–	–	[44]
***P(DMAM-co-DVB)***	123	–	5.62	0.34	–	9.00	36–61	[90]
***PVA/QPECH***	92–118	–	82.35–158.54	0.64–1.76	–	11.0–30.0	24.79–42.24	[76]
***PA-NMP***	–	–	41.02	2.3	0.92	37.0 (6.0 for Fe^2+^)	103.00	[91]
***QPPO/PVA***	163	–	71.9	1.59	–	30.06	43.8	[92]
***DF-120***	320	–	42.00	1.96	–	9.00	18.5	[92]
***Porous DPPO***	0.7–1.4	–	1 51.1 ± 5.3	1.79 ± 0.03	–	66 ± 3	96.9 ± 2.5	[93]
***AMX-134 (Neosepta AFX)***	134	90.0 ± 0.8	26.0 ± 0.20	1.40 ± 0.04	0.533	40	25	[35,37,94]
***DSV***	110	96.0 ± 0.4	110	–	0.526	26	15–18	[35,38,44]
***ACS***	121	94.0 ± 0.4	121	–	0.581	–	–	
***Fuji A***	123	89.0 ± 0.7	123	–	0.519	–	–	
***FumaSep® (Q-SPEEK)***	45–55	85	10.0–25.0	1.6–2.1	–	10–12.22	–	[81,95,96]
***PEEK-Q***	>80	93–98	30.0	0.90	–	10–12.22	–	
***Nafion® 117***	115	–	21.0 ± 1.0	0.90	–	7.84	1.75	

a Measured thickness (using a micrometer screw gauge) prior analysis. Note: ‘α’ is permselectivity. ‘δ’ is thickness; ‘SD.’ is swelling degree; ‘IEC’ is Ion exchange capacity, ‘t^a^_(−)_’ is transport number (of the Cl^−^ Ion) in the membrane. U_d,d_ is the diffusate dialysis coefficient (for H^+^ ions) and ‘S’ is the separation factors.

**Table 6:** Parameters of AEMs related to Mechanical Performance. All membranes were tested at 25 °C (RTP) and 50% RH, unless specified otherwise in parenthesis.

	δ(μm)	σ_B_ (MPa)	ε_B_ (%)	E_t_ (MPa)	Ref.
*QPECH AEM (QP/A-06)*	133^a^	25.5	28.3	329.56–328.84	Present Work
***PEEK-Q***	>80	47.97	23.58	>40	[61,97]
***Brexar 43***	70	19.0 (60 °C)	–	75–400	[73]
***ATMPP***	100	26.0 (60 °C)	–	75–400	
***FumaSep®FAA-30-5 (Q-SPEEK)***	45–55	25.0–40.0	15.0–60.0	40–80	[95]
***Nafion® 115***	115	27.10	225^b^	249	[95,98,99]
***Nafion® 117***	117	27.10	225^b^	249	
***QAFW-10 (Q-CHPTAC-Cel)***	300	60.0 ± 2.2	–	60.02	[100]
***AMX (Neosepta AFN/X)***	140	29.47	15.6	15.0	[101]
***PSU-TMA-OH***	20	20.0	11.0	680	[102]
***PSU-DABCO-OH***	30	24.0	5.0	680	

a Measured thickness (using a micrometer screw gauge) prior analysis. Note: ‘δ' is thickness; ‘σ_B_ ‘is tensile strength/tensility (stress at break); ‘ε_B_’ is elongation at break.

b Supported by PTFE mesh.

**Table 7:** Parameters of AEMs related to resistance and conductivity using different techniques.

	r (Ω.cm)	σ (S/cm)	Type	Method	Ref.
*QPECH AEM (QP/A-06)*	6.411	0.157	DC	Four Probe Co-linear DC Method^a^	Present work
***PECH-C***	6.165 ± 0.185	0.157–0.167	AC	Kroll's Method	[44]
***AM-PP***	1.14 ± 0.05	0.88 ± 0.04	DC	Kroll's Method	[44]
***AMX***	2.35 ± 0.05	0.425 ± 0.005	AC	Kroll's Method	[78]
	2.67	0.524	AC	EIS	
***DSV***	3.07 ± 0.20	0.3255 ± 0.0196	AC	Kroll's Method	
***ACS***	4.39 ± 0.10	0.225 ± 0.05	AC	Kroll's Method	
***Brexar 70***	17.54	0.057	AC	EIS	[73]
***Nafion® 115***	7.41 ± 0.92	0.137 ± 0.17	AC	EIS	
***AMPS (58)-GDMA (42)***	0.77 ± 0.07	0.0132 ± 0.004	AC	EIS	[94]
***PAA-co-PMMA/PVC***	<13.0	0.076	AC	EIS	[103]
***Nafion® 117***	44.0 (Na_2_SO_4_)	0.0222	AC	Chronopotentiometry	[104]
***HDX 100***	60.36 (Na_2_SO_4_)	0.0165	AC	Chronopotentiometry	
***SW/DWCNT-PVA/PES***	7.2 × 10^−5^	13.933	DC	Four Probe Co-linear DC Method^a^	[83]
***PEEK-Q***	90.9	0.011	AC	EIS	[61]
***FumaSep®***	0.6–1.5	0.003–0.008	AC	EIS	[95]
***GO/QPVS-DVB (AEM)***	≤15	0.067	AC	LCR	[100]
***GO/CEM***	≤20	0.050	AC	LCR	

a This analysis is particularly reported for an electrochemically conductive ultrafiltration membrane and has not been reported for moderately conductive AEMs to date. Note that, ‘r’ — Areal resistance (r = R_m_A; where ‘R_m_’ is membrane resistance and ‘A’ is area); ‘σ’ — Conductivity of the membrane.

Moreover, QP/A-06 membrane shows a better mechanical strength, a relatively comparable conductivity and resistance as compare to reported membranes. The membrane synthesized in our present work possesses at about the same thickness as the listed membranes.

### 3.6 Techno-economical feasibility of the synthesized QPECH AEM

#### 3.6.1 Membrane cost/price estimation

QPECH-PAN membranes have the potential for large-scale industrial applications aiming at spent acid regeneration and metal ion recovery. In this section, QPECH-PAN membrane preliminary cost has been evaluated. Material costs were provided by the suppliers and vendors. The labor cost per hour was estimated using the local minimum wage of USD $5.2 (8.5-h day) for skilled workers (Sindh Minimum Wage Act, 2015 [105]). For evaluating the probable commercial price of the QPECH-PAN membrane, a “specific” production cost was considered. This specificity refers to the as-is material costs that were associated with the preparation of the QPECH-PAN membrane. Therefore, it is more practical to assume that total production cost can be estimated with raw material costs constituting as much as 50%–75% of the specific production cost per product [106].

The specific production cost of QPECH AEM (QP/A-06) is USD $5.7 per 100 cm^2^ membrane sheet for a dry membrane thickness of 133 μm. It leads to an anticipated price of approximately 8.6–10.2 USD ($)/100 cm^2^ assuming the 50%–75% margins on the total production cost (see Supplementary Information. S.I., Appendix A, S7). The cost accounting information was developed with assistance of technological suppliers and procuring agencies, Table S7.1. Thus, at this stage, the commercial price for our membrane is evidently competitive and relatively lower than that of commercially available membranes used in similar and related applications. A price comparison of commercial AEMs and prepared QP/A-06 is given in Table 8. It shows the prices in USD $/100 cm^2^ (excluding taxes and freight charges) for commercially available ion exchange membranes used for similar acid and metal ion recovery applications.

**Table 8:** Price comparison of commercial AEMs and the prepared QP/A-06.

Anion Membrane	Price (USD $/100 cm^2^) ^[a]^	Manufacturer	Ref.
Neosepta BP-1	135.0	ASTOM, Japan	[78]
Neosepta AHA,	123.3		
Orion AMX (35 μm, self-supporting)	450.0	Orion Polymer, USA	[108]
PiperION (20 μm, self-supporting)	152.0	Versogen, USA	[109,110]
PiperION (80 μm, self-supporting)	492.2		
Nafion 117 (183 μm, self-supporting) ^[b]^	47.0	Chemours, USA	[111,112]
Nafion 211 (25.4 μm, self-supporting) ^[b]^	30.0		
Nafion 212 (50.8 μm, self-supporting) ^[b]^	32.0		
Nafion 1110 (254 μm, self-supporting) ^[b]^	69.0		
Nafion N424 (180 μm, PTFE-reinforced) ^[b]^	94.0		
Xion PEM-Nafion-1000 (10 μm, self-supporting) ^[b]^	213.5	Xergy Inc, USA	[113,114]
Pention 5 μm (5 μm, self-supporting)	370.0		
Lanxess Sybron Ionac MA-7500 (450 μm, self-supporting)	70.0	Rising Sun Membrane, China PR	[115]
FumaSep FAP-450 (45 μm, self-supporting)	25.0	Fuma-tech GmbH, Germany	[95]
FumaSep FAA-3-50 (50 μm, self-supporting)	22.0		
FumaSep FAA-3-75 (75 μm, self-supporting)	27.0		
**QP/A-06 (133** **μm, self-supporting)**	**8.7**–**10.2**		Present Work

Note: ^[a]^ Commercial price for 10 cm × 10 cm AEMs, as of May 2023. These are the commercial prices associated for these membranes. The QP/A-06 has the forecasted commercial price (calculated in Table S7.1). ^[b]^ Although Nafion membranes are of the cationic-type, their use has also been claimed and industrially adopted in spent acid regeneration and metal recovery [81,82].

In comparison with other current technologies, the broader and large-scale applications of commercial membranes are limited due to its cost and complex reaction conditions involved in the synthesis, which make them unsuitable for commercial viability [81,107]. This work not only presents a relatively comparable recovery performance of the QP/A-06 membrane, but also proposes a low-cost method of the AEM preparation.

#### 3.6.2 Economic feasibility

Experimental results for the QP/A-06 indicate appreciable performance for the DD system and it can prove lucrative for sulfuric acid regeneration from acidic wastewater. The economic feasibility of the proposed QP/A-06 membrane has been studied for the practical applications in spent acid regeneration and metal ion recovery. For the present work, we conducted and economic feasibility assessment based on the work of Jeong et al. (2005) [38]. This feasibility assessment has been conducted for a certain textile manufacturing industry in Karachi, Pakistan. Current strategy to eliminate the hazards and negative impact associated with this waste-water involves neutralization by caustic soda (NaOH), caustic potash (KOH) or slaked lime (Ca(OH)_2_) [29]. This inevitably adds further Environmental, Health & Safety (EHS) concerns, higher operational costs, and sustainability issues.

**Fig. 12:**
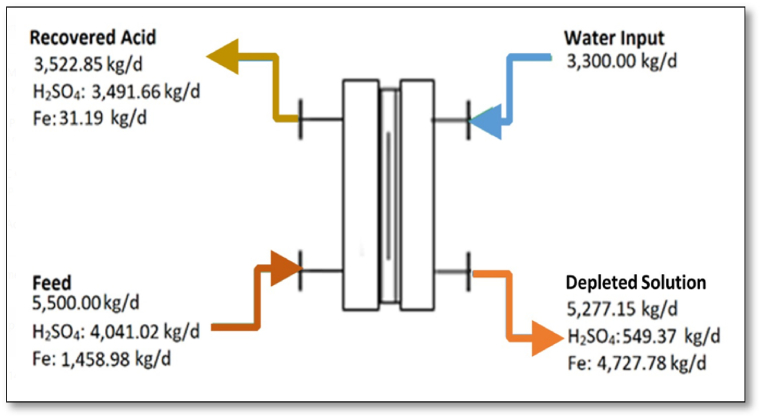
Material balance for acidic wastewater processing (5500 kg/d) in the textile industry.

Textile processing consumes up to 350 kg of water for approximately 100 kg cotton fibre processed [116]. For the present case, the textile industry processes up to 1570 kg/d of textile fabrics i.e., consumes 5500 kg/d water, producing an approximately equal wastewater with acidic and metal content. Using the experimental results of the DD performed with QP/A-06, the material balance can be solved to get Table 9 (Fig. 12). Equation (11), (12), (13), (14) in this regard can be used to calculate the quantity of components in certain streams. The acidic wastewater contains 10.7% acidic content of which 5.6% is free H_2_SO_4_ and about 0.24% consist of Fe^2+^.

**Table 9:** Economic Feasibility of Spent Acid Regeneration and Metal Ion Recovery DD runs, at 5500 kg/d, in the textile industry.

	Water Input (kg/d)^a^	Feed (kg/d)	Spent Solution^b^ (kg/d)	Recovered Acid (kg/d)^c^
	3300.00	5500.00	5277.15	3522.85
**H**_**2**_**SO**_**4**_	–	4041.02	549.37	3491.66^d^
**H**_**2**_**O**	3300.00	1458.98	4727.78	31.19

**Note:** Operation time: 8.5 h, equiv. To one (working) day.

a Feed/Water = 0.6.

b Mass Balance: F + W=R + S.

c Also called ‘Depleted’ Solution.

d Worth US $83,799 of technical-grade H_2_SO_4_ used.

Details regarding the techno-economic analytical considerations for both systems have been provided in the Supplementary Information. S.I., Appendix A, S8. Based on the material balance (see Fig. 12), material input saved for the technical-grade H_2_SO_4_ (90% w/v) and NaOH (50% w/v) are 1,047,497.6 kg (worth US $83,799.8) and 854,402.57 kg (worth $441,726.1) per annum, respectively. Mordant recovery from the spent metal-rich acidic wastewater saves 49,442.95 kg per annum (worth $9888.59). This leads to a total recovered cost of US $535,414.53 per annum. Further, the total capital investment for a commercial-scale acid dialyzer (such as the AP-1500) is typically US$ 500,000 [35,38]. The investment breakdown is provided in Table S5.2.

The investment recovery or break-even point has been estimated to be **11.2 months**. It is evident that the major economic benefits for the spent acid regeneration/recovery are significantly dependent on material cost savings (for NaOH and H_2_SO_4_). Considering that the QP/A-06 possesses an operating life of ≥5 years, the proposed scale-up can bring US $2.63 million profit in addition to improved wastewater discharge management for the textile industry.

A comparative OPEX calculation of the proposed DD with that of the acid neutralization (AN) system in current use at the textile plant has also been performed. The characteristics of the lab-scale DD system using QP/A-06 AEM have been scaled to the proportions of AP-1500 dead-end Acid Recovery Unit (MechChem Associates, Inc., USA; ≥5500 kg/d). The maintenance cost for DD is relatively lower than conventional acid neutralization (AN) unit. Energy consumption and freshwater input is also considerably lower. The details of the two systems are further provided in the Supplementary Information, S.I., S8.2 of this paper. Moreover, the cost of each type of input to the OPEX is tabulated in Table S8.3. Results indicate that DD is an efficient system for spent sulfuric acid regeneration/recycling, offering significant returns and cost-savings. Comparing annual expenses, reveals that the DD system brings about significant cost savings against each of the current AN's expenses. It is to be noted that the DD system does incur additional costs associated with pre-treatment cartridges to improve system performance and operating life. The DD system saves up to **40.91%** of the current OPEX of the AN in use by the textile industry.

#### 3.6.3 Technical feasibility

Incorporation of multiples membrane stack into the chloralkali process, textile, polymer & petrochemical manufacturing process, hydrometallurgical, and mining process can demonstrate many benefits. The experimental results reveal that the use of said AEM in a single stack (single flat membrane plate-and-frame module), results in the following.

(1) DD is a simple unit operation and higher cost savings in terms of energy consumption (only needed for waste pumping) and can run automatically without extensive maintenance for a considerable period.
(2) Originally, the textile waste acid was directly neutralized with NaOH before discharge into an acid storage facility. The proposed technology consumes no such chemicals but may be helpful in the recovery of chemicals. This further reduces the material expenses of the plant, as demonstrated above.
(3) The recovered acid can be recycled back, thus, saving acid consumption in the processes.
(4) Valuable metals (here, Fe2+) and the residual acid can be recovered by recycling the waste stream and can be sent back into the initial stages.
(5) Although at an initial stage, the proposed technology can be optimized to implement “chemical recycling” strategy, yielding significant environmental and economic benefits.
(6) QPECH AEM properties can be finely tuned at optimum ratios of 0.6 and 0.5 PECH/PAN blend and amine ratio respectively, which would yield the membranes possessing the best ion exchanging, mechanical and permselective character.
(7) Practical operations can evidently be enhanced by using multiples of the proposed flat membrane plate-and-frame modules. However, the current single stack plate-and-frame module can be run at the following operating conditions to ensure effective performance: feed flow-rate = 4.2 L/h, flow ratio of demineralized water to feed = 0.6, and operation time of ca. 20–30 min. An 80–90% recovery can likely be achieved with multiple modules.
(8) A steady-state was achieved after 20 min of operation in most tests performed.
(9) At current conditions, the VEF can be helds above low recycling burden levels (VEF <1.0).
(10) The acid recovery and Fe2+ recovery ratio attained for Fe2+ is 48% at 20 min (Si: 84.57) and 72% for acid at 30 min (Si: 126.51), using the QP/A-06 AEM in the single stack plate-frame module. In contrast to other AEMs used for similar experiments with Fe2+-rich acidic wastewater, the recovery is relatively comparable (77–83%) for a single stack module [38,89,117]. It is to be noted that this recovery efficiency will likely increase in multiple stacks for the present membrane.
(11) The recovered acid can be reused for industrial operation. Valuable metals (here, Fe2+) can also be recovered.
(12) Techno-economic evaluation of the QPECH-PAN AEM indicates an attractive short investment recovery of **11.2 months** and a cost-saving on the OPEX by approximately **40.91%** for a select textile industry**.**

## 4 Conclusion

In this study, the AEM was optimized with promising results that would help the industrial sector to save a relatively efficient amount of investment for acidic and metal-rich wastewater treatment and run-offs containing spent acid and metal ions. QPECH-PAN membranes were fabricated by the alignment of 1,4-diazobicyclo[2.2.2]octane as the aminating and cross-linking agent and PAN as the strength modifier to provide efficient mechanical stability and efficiency under the pressure drop in a diffusion dialysis (DD) operation. The prepared QPECH AEM (“QP/A-06”) indicated relatively enhanced mechanical integrity, high acidic separation capability, and high ionic conductivity. The morphological change was verified by the AFM, FTIR imaging and FE-SEM analysis.

The spent acid regeneration and metal recovery QPECH-PAN membrane selection (QP/A-06) was evaluated and its techno-economical attributes were compared with the commercially available AEMs. Experimental investigations were conducted to examine its performance in recovering H_2_SO_4_ and Fe^2+^ from waste acid solution and metal-rich acid tailing. Test runs on the single flat membrane plate-and-frame module, indicate that the QP/A-06 is comparably effective for acid regeneration/recovery (72%) as well as metal ion recovery (48%). The performance of the QP/A-06 will be further enhanced in terms of physiochemical properties and structure for spent acid regeneration and metal ion recovery, in a future paper.

Further, an undisclosed textile industry was considered for the economic feasibility analysis for the use of QP/A-06 in the condition as presented herein in a simulated scale-up (≥5500 kg/d acidic wastewater). Estimations conclude that the QP/A-06 is set to bring a 40.91% saving on the OPEX of an acid neutralization process in place. The forecasted investment recovery point is 11.2 months with US $0.53 million in costs saved for H_2_SO_4_ and NaOH. We believe that said indigenously prepared, low-cost QPECH AEM, as proposed in this paper, would likely play a significant role in “import substitution industrialization (ISI)” in increasingly economically uncertain and stagflation-ridden global markets.

The authors consider that the present work will contribute to addressing the possible knowledge gap in QPECH material development and innovation for improving the sustainability portfolio of the chemical industries across versatile applications. This study proposes a potential scalable, low-energy and cost-effective technology for the recycling of untreated acidic and metal-rich wastewater from (but not limited to) the textile industry.

## Author contribution statement

Shazia Perveen: Conceived and designed the experiments; Wrote the paper. Syed Ghazanfar Hussain: Conceived and designed the experiments. Muzammil Jalil Ahmed: Performed the experiments; Analyzed and interpreted the data. Ruba Khawar, Taha Bin Siraj, Maryam Saleem: Performed the experiments.

## Data availability statement

Data will be made available on request.

## Supplementary information

Additional information regarding procedures and tables is provided in Appendix A, whereas figures regarding certain procedures are provided in Appendix B.

## Funding

This work was funded by the 10.13039/501100004681Higher Education Commission, Pakistan under the Technology Transfer Support Fund program (No: 20-TTSF-130/RGM/R&D/HEC/2020).

## Declaration of competing interest

The authors declare that they have no known competing financial interests or personal relationships that could have appeared to influence the work reported in this paper.
