# Supplementary material for: A Viable and sustainable flat- membrane plate-and-frame module for spent acid regeneration and metal ion recovery
Source: Heliyon. 2023 Jul 17;9(8):e18344. doi: 10.1016/j.heliyon.2023.e18344 (PMC10382638; doi:10.1016/j.heliyon.2023.e18344)
Supplement: Multimedia component 1 [file mmc1.docx]

**Supplementary Information**

A Viable and Sustainable Flat- Membrane Plate-and-Frame Module for Spent Acid Regeneration and Metal Ion Recovery

Shazia Perveen *^a ,*^,* Syed Ghazanfar Hussain *^a^,* Muzamil Jalil Ahmed *^a^*, Ruba Khawar *^a^*, Taha Bin Siraj *^a^*, Maryam Saleem *^a^*.

^a^ *Department of Chemistry, NED University of Engineering & Technology, University Road, Karachi 75270, Sindh, Pakistan.*

^*^ To whom all correspondence should be addressed.

Phone: +92 334 3153430. Fax: N/A. E-mail: [shaziaperveen@neduet.edu.pk](mailto:shaziaperveen@neduet.edu.pk) (Dr. Shazia Perveen, Assistant Professor, Department of Chemistry, NED University of Engineering & Technology, University Road, Karachi 75270, Sindh, Pakistan.)

# Appendix A: Additional Information

## Synthesis of Quaternized Polyepichlorohydrin (QPECH)

Briefly, PECH (11 g) was gradually dissolved in DMSO (32g) to prepare the active polymer solution. The dissolution was conducted under vigorous stirring (1100-1500 rpm) at 30-40℃ for ca. 16 hours. PAN (18.3 g) was dissolved in DMSO (140 g) under stirring at room temperature for ca 4.0 hours to prepare the conditioner polymer solution. DABCO (12.53 g) was dissolved in DMSO (90.0 g) under vigorous stirring (1100-1500 rpm) at 30-40℃ for 3.5 hours, to prepare the conditioner polymer solution. Finally, these three solutions were homogenized in a three-neck round-bottom flask. Notably, a pre-reaction stage was conducted in a paraffin oil bath at 80^o^C for 30 min. The pre-reacted casting solution obtained, at the fixed blend ratio, is of a carmine (golden to dark red) color.

## Quaternized Polyepichlorohydrin Polyacrylonitrile (QPECH-PAN) Self-supporting Asymmetric Membrane Preparation

Post-synthesis, a specific amount of the pre-reacted casting solution was spread over a 10 cm × 10 cm glass plates using a syringe. A membrane coater (equipped with the doctor blade) was used to uniformly cast the membrane over the 10 cm × 10 cm glass plates. The glass plate was subsequently placed in a hermetic glass box to prevent further DABCO evaporation. After this, the hermetic glass box was placed in an oven (Pol-Eko Aparatura SLN 53, Poland) for 2 hrs. at 110^o^C. The amination and cross-linking are made to occur at this stage. Next, the seal was removed to allow controlled residual solvent evaporation at 130^o^C for 30 min. After cooling, the polymer film was soaked in 0.5M NaCl_(aq)_, from which it peels off from the glass substrate and stored in the same solution.

## S3. Physico Chemical Characterization

### S3.1. Ion Exchange Capacity (IEC):

the IEC of the synthesized QPECH-PAN membrane(s) was determined via an argentometric titration method. Briefly, the membrane was soaked in 0.5M NaCl_(aq)_ for at least 15 hrs, followed by rinsing with deionized water and soaking in aqueous Na_2_SO_4_ (0.25M) for >3 hrs to undergo ion exchange (i.e., Cl^-^ substitution by SO_4_^2-^). The sample solution was then titrated with aqueous AgNO_3_ (0.2M) using K_2_CrO_4(aq)_ indicator. The end-point is indicated by the appearance of reddish color precipitates of AgCrO_4(ppt)_. IEC values (mmol/g _dry membrane_) were calculated by:

$IEC =\frac{C_{{AgNO}_{3}}V_{{AgNO}_{3}}}{W_{dry}}$*; (eq. 1)*

Where V_AgNO3_ and C_AgNO3_ are the volume and concentration of consumed AgNO_3_ solution respectively, W_dry_ is the dried membrane mass.

### S3.2. Swelling Degree (SD) and other parameters:

For SD measurement, the synthesized QPECH-PAN membrane was soaked in deionized water for at least 24 hours. The membrane was dried (by removing surface water using cotton wipes) and weighed. Subsequently, the wet membrane was dried at 30℃ in a drying oven (Pol-Eko Aparatura SLN 53, Poland) for 6-8 hrs. until a constant dry weight was achieved. SD was calculated using:

$SD=100\frac{m_{wet}-m_{dry}}{m_{dry}};$ *(eq. 2)*

Where, m_wet_ and m_dry_ are the mass of the wet and the dry QPECH-PAN membrane, respectively. Other parameters such as bound water per ionic group ‘λ’, membrane void porosity ‘ε’ (volume of free solution within the membrane per unit volume of wet membrane) and fixed-charge density ‘FCD’, were also calculated using the parameters of eq. 10, 11 and 12 respectively.

$\lambda\boldsymbol{=}\frac{SD}{M_{H_{2}O}\times IEC}$ *(eq. 3)* [2,3]

$\boldsymbol{\varepsilon}\boldsymbol{=}\left( 1+\frac{\rho_{i}}{SD\cdot\rho_{m}} \right)^{-1};$ *(eq. 4)* [2,3]

$FCD=\frac{IEC}{SD};$ *(eq. 5)* [2,3]

Where, ‘M_H2O_’ is the molar mass of a water molecule (18.0 g/mol), ‘ρi’ and ‘ρm’ is the density of absorbed water (0.997 g/cm^3^) and dry membrane respectively.

## S4. DD Acid/Metal recovery Titrimetric Data

Titrimetric analysis was performed after every 15 minutes to determine the amount of acid and/or metal in the targeted sample. Conventional acid-base titrimetric method was adopted for quantitative analysis of simulated acid wastewater samples ‘SS/A-01’ (1.05 M H_2_SO_4_) using 2M NaOH as the titrant. The mole ratio of SO_4_^2-^ ions to OH^-^ ions is 1:2 according to the following chemical relation:

2NaOH_(aq)_ + H_2_SO_4(aq)_ → Na_2_SO_4(aq)_ + 2H_2_O

Permanganate redox titration was adopted for quantitative analysis of simulated metal-rich acidic wastewater sample ‘SS/M-02’ (0.03 M FeSO_4_.7H_2_O) using 0.1M KMnO_4_ as the titrant. The mole-ratio of MnO_4_^-^ to Fe^2+^ is 1:5, according to the following chemical reaction:

8H^+^_(aq)_ + MnO_4_^-^ _(aq)_ + 5Fe^2+^ → Mn^2+^ _(aq) +_ 5Fe^3+^ _(aq)_ + 4H_2_O

**Table S4.1.** Tabulated data of DD Acid/Metal recovery by the single flat membrane plate-and-frame module using the prepared QPECH AEM.

|  | ***SS/A-01***  *0.105M H_2_SO_4_* | | | |  | ***SS/M-01***  *0.03M FeSO_4_.7H_2_O/H_2_SO_4_* | | | |
| --- | --- | --- | --- | --- | --- | --- | --- | --- | --- |
|  | ***Diffusate*** | | ***Retentate*** | |  | ***Diffusate*** | | ***Retentate*** | |
| **Time (min)** | **Conc./ (g/L)** | **pH** | **Conc./ (g/L)** | **pH** |  | **Conc./ (g/L)** | **pH** | **Conc./ (g/L)** | **pH** |
| 10 | 14.7 | 1 | 102.9 | 7 |  | 0.425 | 7 | 6.836 | 7 |
| 20 | 118.58 | 1 | 98 | 6 |  | 4.497 | 6 | 5.317 | 7 |
| 30 | 120.54 | 1 | 95.06 | 6 |  | 1.944 | 5 | 8.355 | 7 |
| 40 | 115.64 | 1 | 93.1 | 5 |  | 1.155 | 5 | 6.076 | 7 |

Note: SS/x-n, where “SS” is the simulated wastewater containing acid “A” or metal “M”. Whereas, “n” is the sample no. corresponding to different concentrations.

## S5. Roughness Parameters for AFM Analysis

**Table S5.1.** Main roughness parameters, as defined and classified by ISO 25178 for 3D surface AFM images calculated for the different images in **Fig 5-6**.

|  | **Fig. Ref.** | **Scan area (μm^2^)** | **Height Parameters** | | | | | | | **Comments** |
| --- | --- | --- | --- | --- | --- | --- | --- | --- | --- | --- |
|  |  |  | **Sq (nm)** | **Sku** | **Ssk** | **Sp (nm)** | **Sz (nm)** | **Sv (nm)** | **Sa (nm)** |  |
| **PAN/ QPECH AEM** | 6a | 1.0 × 1.0 | 4.58 | -0.662 | 3.74 | 13.5 | 38.4 | 24.9 | 3.57 | *Smooth film* |
|  | 6b | 2.0 × 2.0 | 4.91 | -0.421 | 3.57 | 19.9 | 44.3 | 24.4 | 3.86 |  |
|  | 6c | 3.0 × 3.0 | 8.07 | -0.467 | 3.13 | 31.3 | 65.7 | 34.4 | 6.42 |  |
|  | 6d | 5.0 × 5.0 | 10.4 | -0.283 | 2.94 | 31.8 | 76.2 | 44.4 | 8.45 |  |
|  | 6e | 6.2 × 6.2 | 13.3 | -0.123 | 2.76 | 39.9 | 84.3 | 44.4 | 10.7 |  |
|  | 6f | 10.0 × 10.0 | 16.0 | -0.502 | 3.88 | 50.4 | 135 | 84.3 | 12.5 |  |

Note: ‘Sq’ ‘Ssk’ and ‘Sku’ are the root mean square height, the skewness, and the kurtosis of the height distributions respectively. Further, ‘Sp’, ‘Sz’, ‘Sv’ and ‘Sa’ are the maximum peak height, maximum height, maximum pit height and the arithmetic mean height respectively.

## S6. Sheet resistances and Conductivities

**Table S6.1.** Measured sheet resistances and conductivities at constant current (1 mA DC) for a test PAN/QPECH AEM membrane of area 1 × 1 mm^2^ and 133 μm thickness:

| **ΔV^a^** | **R^b^ (Ω/sq)** | **ρ_s_^c^ (Ω.cm^2^)** | **S^d^ (S/cm^2^)** |
| --- | --- | --- | --- |
| 0.128822327 | 128.8223205 | 583.8227567 | 0.00776263 |
| 0.46969986 | 469.6998373 | 2128.679663 | 0.002129019 |
| 0.604085922 | 604.0858935 | 2737.71727 | 0.001655394 |
| 0.528022766 | 528.022741 | 2392.999062 | 0.001893858 |
| 0.514442444 | 514.4424194 | 2331.453045 | 0.001943852 |
| 0.469655991 | 469.6559683 | 2128.480848 | 0.002129218 |
| 0.467260361 | 467.2603385 | 2117.623854 | 0.002140135 |
| 0.436338425 | 436.338404 | 1977.485647 | 0.002291799 |
| 0.436424255 | 436.4242346 | 1977.874631 | 0.002291348 |
| 0.412851334 | 412.851314 | 1871.042155 | 0.00242218 |
| Average: | **R_avg_ = 482.0868** | ρ_s,avg_ = 2184.8174 | **S_avg_ = 0.00210** |

Note: ^a^ Potential difference, ΔV = V_SMU3_-V_SMU2,_ ^b^ Sheet resistance ‘R’, ^c^ Sheet resistivity ‘ρ_s_’, ^d^ Sheet conductivity. The sheet resistance unit ‘Ω/sq’ is dimensionally equivalent to Ω.

## S7. Price Accounting Information

**Table S7.1.** Price accounting information and data resources for QP/A-04 production.

| **No.** | **Name of Input** | **Unit price**  **(USD $/unit)** | **Manufacturer/ Consignor** | **Data Source** |
| --- | --- | --- | --- | --- |
| 1. | Poly(epichlorohydrin) | 16.71/1g | VladaChem GmbH, Germany | Tech. Supplier |
| 2. | Poly(acrylonitrile) | 60.97/1g | Merck-Millipore KGaA | Tech. Supplier |
| 3. | 1,4-diazabicyclo[2.2.2]octane | 7.93/1g | Merck-Millipore KGaA | Tech. Supplier |
| 4. | Dimethyl sulfoxide | 0.39/1mL | Merck-Millipore KGaA | Tech. Supplier |
| 5. | Dimethyl Formamide | 0.14/1mL | VWR Chemicals (RDH/BDH) | Tech. Supplier |
| 6. | Labour cost | 4.65/1hr | Karachi, Sindh, Pakistan | Sindh Minimum Wages Act, 2016 [4] |
| *QP/A-04 Membrane Casting Dispersion* | | 63.3/1mL |  | |
| *Specific Production Cost* | | USD $15.63 | For, 1 ea., 100 cm^2^, 133 μm QP/A-04 AEM | |
| *Production Cost (50%)* | | USD $8.72 |  |  |
| *Production Cost (75%)* | | USD $10.17 |  |  |

## S8. Techno-Economic Feasibility Assessment

### S8.1. Costs and Material Quantities Recovered:

The techno-economic feasibility of the QP/A-04 membrane in a conventional diffusion dialyzer (DD) system has been studied for the textile industry, considering a scaled-up version (AP-1500, MechChem Associates Inc., USA). TEFA has been conducted based on the work of Jeong et al. (2005)[5]. The estimates have been tabulated in **Table S5.1.** Considering that the selected textile industry uses an Acid Neutralizer unit, the cost savings in relation to this process can be estimated. The neutralization reaction is as under. The cost recovery of the mordant, FeSO4.7H2O used in the textile processes has also been included.

$$H_{2}SO_{4}+2NaOH \to Na_{2}SO_{4}+2H_{2}O$$

Table S8.1. Recovery estimates in terms of material quantities and costs.

| **Component** | **Recovered** | | | |
| --- | --- | --- | --- | --- |
|  | **Material** | | **Cost** | |
|  | **(kg/d)** | **kg^1^** | **USD $/kg** | **USD $ ^2^** |
| H_2_SO_4_ (90% w/v) | 3,491.66 | 1,047,497.56 | 0.26 | 83,799.80 |
| NaOH (50% w/v) | 2,848.01 | 854,402.57 | 0.52 | 441,726.13 |
| FeSO_4_.7H_2_O ^3^ | 164.81 | 49,442.95 | 0.45 | 9,888.59 |
| **Total:** | | | | 535,414.53 |

**Note:** ^1^ for 300 days. ^2^ H_2_SO_4_ (90% w/v, technical grade) and NaOH (50% w/v, technical grade) costs USD $0.26/kg and $0.52/kg. ^3^ Mordant.

Table S8.2. Investment cost breakdown for APA-1200 (MechChem Associates Inc., USA; 5,500 kg/d capacity).

|  | **Cost (USD $)** |
| --- | --- |
| Diffusion Dialysis Unit (AP-1500)^1^ | 300,000 |
| Auxiliaries (circuits, pumps, tanks and valves^2^ | 150,000 |
| Electricity, Maintenance, Labour and Other Costs ^3^ | 50,000 |
| **Total** | 500,000 |

**Note:** ^1^ From technical supplier, ^2-3^ Zhang et al. (2020)[6]

Based on the costs recovered and an investment worth USD $500,000 (see breakdown below) for procuring, operating and maintaining the AP-1500 acid dialyzer unit [5,6], the write-off (investment recovered) has been estimated to be **11.2 months** for a year. The write-off is the ratio between investment cost and recovered costs times 12 months.

### S8.2. OPEX Estimation and Comparison with AN:

The spent acid neutralizer (AN), in its current operation, utilizes technical-grade caustic soda (50% NaOH) for neutralizing the acidic wastewater from various textile processes (especially, bleaching). Additionally, there will be precipitates generated at the end of the AN process, which incur costs associated with solid waste removal and disposal. In both cases, consider the regeneration/recovery of 93% wt. H_2_SO_4_ is consumed in the textile manufacturing process. The acid-base neutralization is as under:

$$H_{2}SO_{4}+2NaOH \to Na_{2}SO_{4}+2H_{2}O$$

Additionally, a pre-treatment cartridge (10 µm) has been considered for the spent acid to maintain the QP/A-04 operational cycle and the impact of impurities on the DD process. It is to be noted that the DD process in this estimation, has considered the acid recovery ratio established in Section 3.5 of the manuscript. The OPEX cost breakdown for the AN process in use by the selected textile industry is provided in Table S5.2, in addition to the DD process. Note that, for DD, the costs associated with water consumption, 90% w/v H_2_SO_4_ and 50% w/v NaOH have been estimated from the results of the material balance.

Table S8.3. Comparative OPEX estimates in terms of the AN and DD processes.

| **Items** | **Annual OPEX** | | | | |
| --- | --- | --- | --- | --- | --- |
|  | **Unit** | **Cost** | **AN ^[a]^** | **DD ^[b]^** | **Savings** |
| Electricity ^[c]^ | USD$/kWh | 0.15 | 1,734.2 | 888.0 | 846.2 |
| Fresh Water ^[d]^ | USD$/L | 4.40 | 24.2 | 14.5 | 9.7 |
| 90% w/v H_2_SO_4_ ^[e]^ | USD$/kg | 0.26 | 147.3 | 142.8 | 4.5 |
| 50% w/v NaOH ^[e]^ | USD$/kg | 0.52 | 1,424.5 | 738.2 | 686.4 |
| Solid Waste Disposal ^[f]^ | USD$/kg | 0.09 | 6.7 | 14.1 | (7.4) |
| Man-power ^[g]^ | USD$/hr | 5.20 | 1,560.0 | 1,560.0 | - |
| Maintenance and Other Costs ^[h]^ | USD$ |  | 30,000.0 | 15,000.0 | 15,000.0 |
| Pre-Treatment Cartridge ^[i]^ | USD$/pc | 6.60 | - | 2,262.6 | (2,262.6) |
| **Total** | | | 34,896.9 | 20,620.2 | **14,276.7** |
|  | | |  |  |  |
| **%Saving on OPEX:** | | | **40.91** | | |

**Note:** All costs as of 5^th^ May 2023

^[a]^ Acid Neutralizer Unit (three 94.71 m^3^/d capacity batch reactors). Also reported elsewhere [7]

^[b]^ Diffusion dialyzer (150 m^2^) using QP/A-04.

^[c]^ Commercial rates for electricity respective of Karachi, Pakistan [8]

^[d]^ Commercial rates for freshwater respective of Karachi, Pakistan [9]. Base rate: US $22 per 5000 litres.

^[e]^ Inflation-adjusted. From technical supplier

^[f]^ Inflation-adjusted. Also reported elsewhere [10].

^[g]^ For an 8.5 hr working day for an operation year, at US $5.2 as per Sindh Wages Act (Sindh Government, 2015)[4].

^[h]^ Inflation-adjusted. Also reported elsewhere [6].

^[i]^ Inflation-adjusted. Also reported elsewhere [10].

# Appendix B: Images and Figures

## DC Resistivity

The four-probe co-linear method or Kelvin technique uses one probe as a source current, another as a ground (or secondary source) and the remaining two probes for measuring the potential drop. This technique involves bringing four equidistant probes in contact with a material of unknown resistance. . An electric current of 1 mA DC is applied to SMU1, while SMU3 and SMU2 measure the generated potential (the difference between the two is measured). SMU4 is grounded. A 1 nA DC is applied to SMU3 by default configuration. The four probes eliminate measurement errors due to the probe resistance, the spreading resistance under each probe, and the contact resistance between each metal probe and the test material (QPECH AEM). Asa high-impedance voltmeter draws little current, the voltage drop measured by it is very small [1].


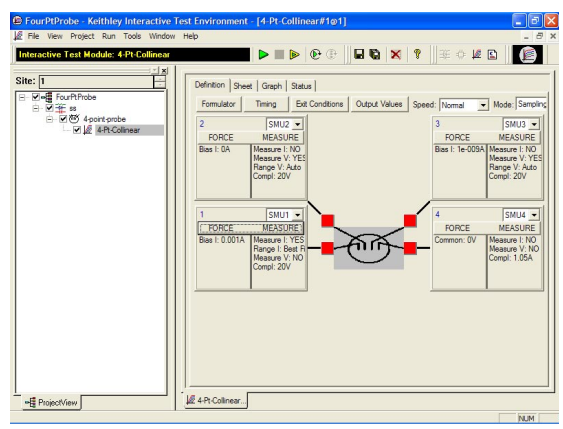


Fig. S2. The Keithley Interactive Test System (KITE) software configuration for the four probes co-linear bulk resistivity and conductivity analysis.

**Fig**

**Fig S3. (a)** The Everbeing EB-6V mmW Optimized Probe station (Everbeing Int’l Corporation, Taiwan) was used for the four probes’ co-linear bulk resistivity and conductivity analysis. **(b)** shows the four-probe system (collinear arrangement not shown).

## FE-SEM Image-based Porosimetry

The MatLab Code for Rabbani’s work can be found at: (<https://www.mathworks.com/matlabcentral/fileexchange/70245-sem-image-porosity-and-pore-size>). The Pore size estimations using this code for the FESEM images in the paper are provided below:


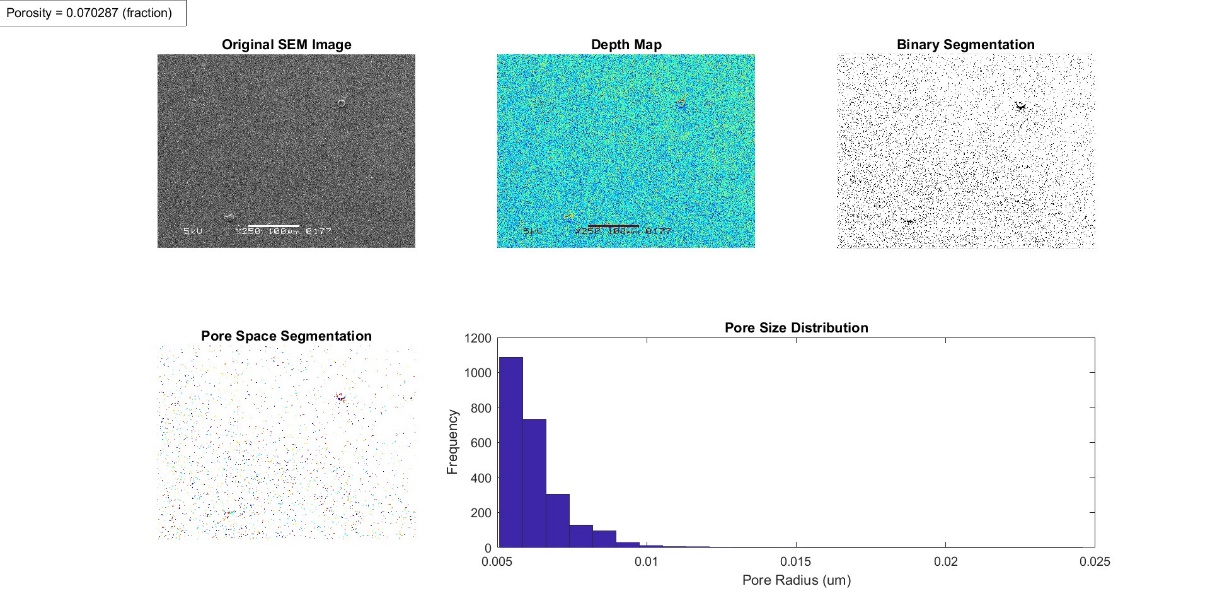


Fig. S1(a). Surface image (Fig 7a; at 250x) processed using the *SEM Image Porosity & Pore Size* semi-automated image processing algorithm (porosity: 0.070287)


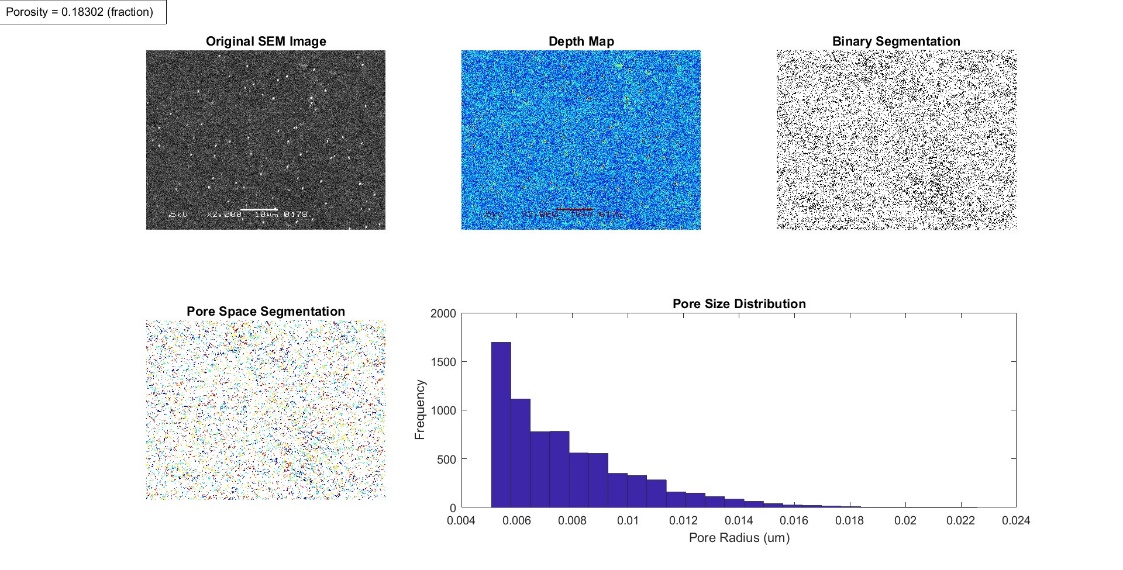


**Fig. S1(b).** Surface image (Fig 7c; at 2,000x) processed using the *SEM Image Porosity & Pore Size* semi-automated image processing algorithm (porosity: 0.18302)


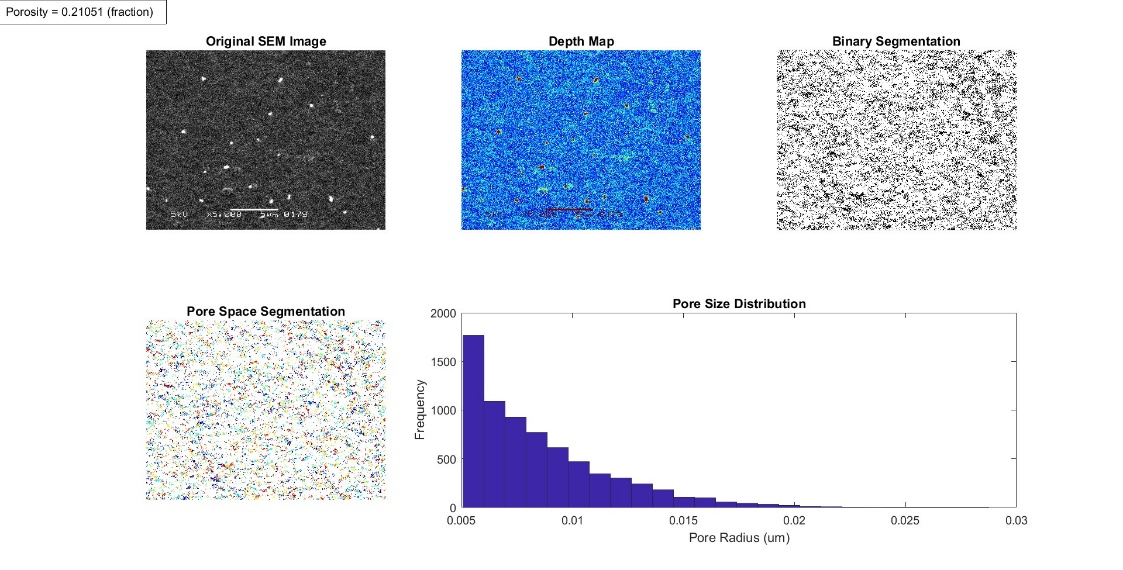


**Fig. S1(c).** Surface image (Fig 7e; at 5,000x) processed using the *SEM Image Porosity & Pore Size* semi-automated image processing algorithm (porosity: 0.21051)


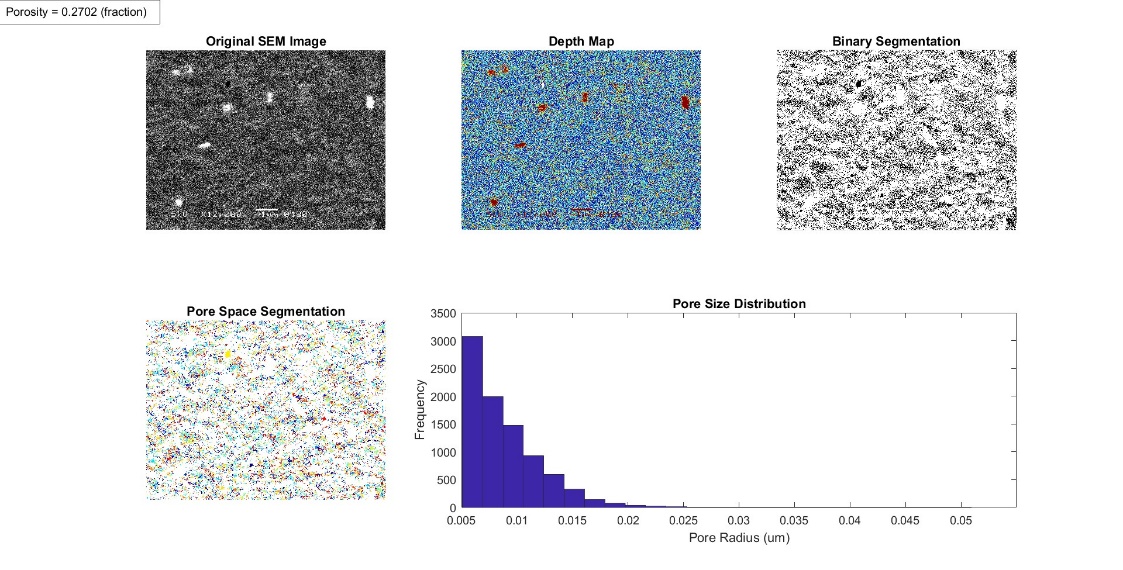


**Fig. S1(d).** Surface image (Fig 7g; at 12,000x) processed using the *SEM Image Porosity & Pore Size* semi-automated image processing algorithm (porosity: 0.2702)

# References

[1] Keithley Instruments, Four-Probe Resistivity and Hall Voltage Measurements with the Model 4200-SCS, Application Note Series, Number 2475. (2004).

[2] T. Sata, G.N. Jones, T. Sata, Ion Exchange Membranes: Preparation, Characterization, Modification and Application, Ion Exchange Membranes. (2004). https://doi.org/10.1039/9781847551177.

[3] T. Xu, Ion exchange membranes: State of their development and perspective, J Memb Sci. 263 (2005) 1–29. https://doi.org/10.1016/J.MEMSCI.2005.05.002.

[4] Sindh Government Pakistan, Pakistan - Sindh Minimum Wages Act, 2015 (Sindh Act No.VIII of 2016)., Legislation. (2016). https://www.ilo.org/dyn/natlex/natlex4.detail?p_isn=102145 (accessed May 7, 2023).

[5] J. Jeong, M.S. Kim, B.S. Kim, S.K. Kim, W.B. Kim, J.C. Lee, Recovery of H2SO4 from waste acid solution by a diffusion dialysis method, J Hazard Mater. 124 (2005) 230–235. https://doi.org/10.1016/J.JHAZMAT.2005.05.005.

[6] C. Zhang, W. Zhang, Y. Wang, Diffusion Dialysis for Acid Recovery from Acidic Waste Solutions: Anion Exchange Membranes and Technology Integration, Membranes 2020, Vol. 10, Page 169. 10 (2020) 169. https://doi.org/10.3390/MEMBRANES10080169.

[7] D. Sharma, A. V. Karre, K.T. Valsaraj, S. Sharma, Intensification of a neutralization process for waste generated from ion exchange regeneration for expansion of a chemical manufacturing facility, Processes. 9 (2021). https://doi.org/10.3390/PR9081285.

[8] Karachi Electric (KE), Tariff Structure - K-Electric, Press Release. (2021). https://www.ke.com.pk/customer-services/tariff-structure/ (accessed May 7, 2023).

[9] ARY News, KWSB jacks up water tanker rates in Karachi, News Article. (2023). https://arynews.tv/water-tanker-rates-jacked-up-in-karachi/ (accessed May 7, 2023).

[10] A. Merkel, A.M. Ashrafi, M. Ondrušek, The use of electrodialysis for recovery of sodium hydroxide from the high alkaline solution as a model of mercerization wastewater, Journal of Water Process Engineering. 20 (2017) 123–129. https://doi.org/10.1016/j.jwpe.2017.10.008.
